# Assessment of facility and health worker readiness to provide quality antenatal, intrapartum and postpartum care in rural Southern Nepal

**DOI:** 10.1186/s12913-019-4871-x

**Published:** 2020-01-06

**Authors:** Tsering P. Lama, Melinda K. Munos, Joanne Katz, Subarna K. Khatry, Steven C. LeClerq, Luke C. Mullany

**Affiliations:** 1 Department of International Health, Johns Hopkins Bloomberg School of Public Health, 615 N. Wolfe Street, Suite W5009C, Baltimore, MD 21205 USA; 2 Nepal Nutrition Intervention Project – Sarlahi (NNIPS), Kathmandu, Nepal

**Keywords:** Quality of care, Facility Readiness, Maternal care, Newborn care, Health worker knowledge, Nepal

## Abstract

**Background:**

Increased coverage of antenatal care and facility births might not improve maternal and newborn health outcomes if quality of care is sub-optimal. Our study aimed to assess the facility readiness and health worker knowledge required to provide quality maternal and newborn care.

**Methods:**

Using an audit tool and interviews, respectively, facility readiness and health providers’ knowledge of maternal and immediate newborn care were assessed at all 23 birthing centers (BCs) and the District hospital in the rural southern Nepal district of Sarlahi. Facility readiness to perform specific functions was assessed through descriptive analysis and comparisons by facility type (health post (HP), primary health care center (PHCC), private and District hospital). Knowledge was compared by facility type and by additional skilled birth attendant (SBA) training.

**Results:**

Infection prevention items were lacking in more than one quarter of facilities, and widespread shortages of iron/folic acid tablets, injectable ampicillin/gentamicin, and magnesium sulfate were a major barrier to facility readiness. While parenteral oxytocin was commonly provided, only the District hospital was prepared to perform all seven basic emergency obstetric and newborn care signal functions. The required number of medical doctors, nurses and midwives were present in only 1 of 5 PHCCs. Private sector SBAs had significantly lower knowledge of active management of third stage of labor and correct diagnosis of severe pre-eclampsia. While half of the health workers had received the mandated additional two-month SBA training, comparison with the non-trained group showed no significant difference in knowledge indicators.

**Conclusions:**

Facility readiness to provide quality maternal and newborn care is low in this rural area of Nepal. Addressing the gaps by facility type through regular monitoring, improving staffing and supply chains, supervision and refresher trainings is important to improve quality.

## Background

Despite declining maternal mortality ratio (MMR) and neonatal mortality rates (NMR) and increasing rates of facility deliveries, an estimated 303,000 maternal deaths [1], 2.7 million neonatal deaths [2], and 2.6 million stillbirths occurred in 2015 worldwide [3]. While the recent focus on the Millennium Development Goals (MDG) target for maternal mortality reductions aided the scale-up of effective interventions, only 9 of 75 high burden “countdown” countries achieved their targets for maternal mortality [1]. Even more ambitious are the Sustainable Development Goals (SDG) of reducing MMR and NMR to 70/100,000 livebirths and 12/1000 livebirths, respectively, by 2030 [4]. Increasing universal coverage of delivery in health facilities is necessary but insufficient to meet these targets; if quality of care (QoC) provided is poor, improved maternal and neonatal health outcomes are unlikely [5–7]. Recent high-profile series in *The Lancet* [8, 9], Midwifery [10, 11], and Maternal Health [12, 13] highlight substantive variability in mortality risk within facilities in low middle income countries [5, 14], and emphasize the critical need to measure QoC indicators and improve quality of services around the time of birth. The 2018 *Lancet Global Health Commission* on high-quality health systems (HQHS) in the SDG era asserts that providing health services without guaranteeing a minimum level of quality is ineffective, wasteful and unethical [15]. This Commission estimates that poor-quality care resulted in 82 deaths per 100,000 people in LMIC and that high-quality health systems could save more than 8 million lives each year in low- and middle- income countries (LMICs) [15]. Maternal and newborn deaths are a particularly sensitive measure of health system quality, because many deaths stemming from labor complications can be averted with appropriate treatment [16].

Although greatly reduced from 539 in 1996, the MMR in Nepal remains high at 239 maternal deaths per 100,000 live births and NMR has decreased from 33 in 2006 and 2011 to 21 deaths per 1000 live births in 2016 [17]. Additionally, the Government of Nepal has expanded 24/7 delivery sites like birthing centers (BCs), and basic and comprehensive emergency obstetric and newborn care services (BEmONC, CEmONC) at existing primary and secondary level health facilities and hospitals [18].

The rapid expansion of the Nepal Safe Motherhood Program (NSMP) nationwide likely contributed to an increase in institutional deliveries to 57% in 2015 from 9% in 1996 [17], but this increased demand for services in rural birthing centers might be outpacing the distribution of skilled birth attendants (SBAs) or the supply of necessary medicine and commodities [19]. In this context, we aimed to assess the facility readiness and health worker knowledge concerning of maternal and newborn care that are vital components of health facilities’ capacity to provide quality maternal and newborn care.

## Methods

### Study setting

Data were collected from facilities and providers in Sarlahi district (population ~ 750,000) located in the central southern plains of Nepal, bordering India. The annual birth cohort is approximately 18,000 [18]. Under Nepal’s recently implemented federal system of governance, Sarlahi district falls within Province 2, which has the second-lowest rate of institutional delivery (45%) and the lowest proportion of ANC visits during the recommended months of pregnancy (36%) [17]. In terms of the 2011 Human Development Index (HDI), Sarlahi district has the second-lowest HDI category at 0.402 (national HDI 0.458), thus representing one of the socially and economically disadvantaged population in Nepal [20].

### Study procedures

QoC has various definitions and defined differently by various experts and institutions. The Institute of Medicine (IOM) defines quality as “the degree to which health services for individuals and populations increase the likelihood of desired health outcomes and are consistent with current professional knowledge” [21]. Under the Donabedien framework [22], assessment of the quality of health care is defined as “determining whether what is already known to be the best care is being implemented”; this classic framework comprises three elements: structure, process and outcome [22]. In this study we used the Donabedian framework to assess the structure component of QoC [23], which includes the availability of skilled health workers and a well-functioning health facility. For this study, we used 1) a birthing center audit tool to assess facility accessibility and readiness to provide care with respect to infrastructure, medicines, and supplies/equipment, and 2) a health worker knowledge assessment tool adapted from instruments previously utilized in the national Nepal Birthing Center Assessment of 2013 [24]. These were initially developed through USAID-funded Maternal and Child Health Integrated Program (MCHIP) QoC surveys, which have been implemented in numerous locations globally [25]. Adaptations reflected local interest and importance, which emerged through consultations with key maternal and newborn care stakeholders in the District Public Health Office in Sarlahi, such as how placenta is disposed of, the levels of staff positions for human resources, and the addition of a section on the availability/observation of various types of health facility records/posters (Additional file 1).

After pilot testing the tools in April 2016, we conducted a cross-sectional study on all public and private health facilities that were classified as birthing centers in Sarlahi district between May 4 and August 29, 2016. Through direct observation of the facility and interviews with staff / in-charges, the facility audit focused on the infrastructure, utilities, furniture, medical equipment, and drugs allocated for antenatal care (ANC), labor and delivery, and newborn care services, as well as information on the human resources. All staff (doctors, nurses and auxiliary nurse midwives) engaged in provision of ANC, labor/delivery care, and/or immediate newborn care, were eligible for the health worker interview. Questions focused on work experience, training, and knowledge of maternal (actions to be taken for management and prevention of various maternal complications and ability to make correct diagnoses based on a case scenario illustrating severe pre-eclampsia) and newborn (immediate care practices, signs of severe infections) health. Data were recorded using the Research Electronic Data Capture (REDCap) application on a password-encrypted mobile android device. When possible, the birthing center audit and health worker interviews were completed on the same day; in about half of the facilities, some eligible health workers were not met on the first visit (due to personal or professional leave) but were conducted subsequently upon their return. The lead author, a native Nepali speaker, conducted all the interviews and facility assessment in Nepali using a Nepali translation of the Health worker interview tool as a guide.

### Data analyses

We conducted descriptive analyses of the infrastructure and utilities (physical space, electricity, water supply, toilet, cleanliness), human resource availability, privacy, and capacity to 1) perform the seven signal functions under BEmONC (Table 1) [26] and 2) provide basic ANC services (routine urine and blood tests, tetanus toxoid vaccination, iron/folic acid tablet distribution, etc.), based on the availability and functional status of essential supplies, equipment, and medicines. We coded each BEmONC signal function item as 1 (available and functional) or 0 (not available or functional) and computed an unweighted average of the items to obtain a readiness score for each signal function and facility [27, 28]. The mean scores for each signal function were summarized overall and by facility type: District hospital, primary health care center (PHCC), health post (HP) and private facility. Tests of statistical significance were not conducted given the small number of facilities (*N* = 24).

**Table 1:** Items included in the mean percentage score calculation for the seven BEmONC signal functions

	Signal Function	Items included
1.	Removal of retained products of conception	Manual vacuum aspiration (MVA) or dilation and curettage kit; injectable oxytocin; syringes and needles; and IV solution (ringer’s lactate, dextrose 5% in normal saline (D5NS) or normal saline (NS) infusion)
2.	Parenteral antibiotics for infection	Injectable ampicillin or gentamycin; syringes and needles; and IV solution (ringer’s lactate, D5NS or NS infusion)
3.	Parenteral oxytocin	Injectable oxytocin; syringes and needles; and IV solution (ringer’s lactate, D5NS or NS infusion)
4.	Parenteral magnesium sulphate	Injectable magnesium sulfate; syringes and needles; and IV solution (ringer’s lactate, D5NS or NS infusion)
5.	Manual removal of placenta	Injectable ampicillin; injectable oxytocin; syringes and needles; and IV solution (ringer’s lactate, D5NS or NS infusion)
6.	Assisted vaginal delivery	ventouse (vacuum extractor manual or electrical)^a^
7.	Newborn resuscitation	Bag and mask (infant size), and resuscitation table for newborn

^a^ not assessed in terms of “ever and recent performance”

Elements of health worker knowledge were grouped into domains corresponding to interventions (e.g., PMTCT, AMTSL, etc.), the mean percentage of correct answers was calculated within each domain, and mean scores were stratified by facility type. Chi-squared or Fisher’s exact tests were used to compare health worker scores across facility types and by whether or not the respondent had received the Ministry of Health and Population (MoHP)-sponsored two-month skilled birth attendant (SBA) training. Stata version 13.0 (StataCorp, College Station, TX, USA) was used for all analyses.

### Ethical Approval

The Johns Hopkins Bloomberg School of Public Health Institutional Review Board (Baltimore, USA) and the Nepal Health Research Council, Ministry of Health and Population (Kathmandu, Nepal) reviewed and approved this study. Written informed consent was obtained from health workers prior to the interview.

## Results

### Health Facility Readiness

We included all 22 public facilities with a designated birthing center, reflecting the three tiers of district-level service (1 District hospital, 5 PHCCs, and 16 HPs) [29] and 2 private facilities (1 NGO-run clinic, and 1 community-hospital).

#### Accessibility

All facilities were either directly linked (21 of 24) or within 5 min’ walk (3 of 24) to a road accessible to 4-wheeled vehicles. Six facilities (District hospital, both private clinics, and 3 HPs) had a functional ambulance with fuel, while 1 PHCC and 3 HPs had a non-functioning ambulance.

#### Availability, infrastructure, cleanliness

While all facilities reported providing 24/7 delivery services (except one HP-level birthing center where no auxiliary nurse midwives (ANMs) were posted), a separate room for night shift staff was available in only 18 facilities. Given the frequency of blackouts, backup electrical capacity is required to prevent gaps in quality services; four HPs had no such backup (i.e. neither generator, solar nor inverter). Except for one PHCC that was renting space and had only one room dedicated for delivery, ANC, and postnatal services, all other facilities had a single room with visual and auditory privacy for delivery care. Ten facilities had a separate dedicated space for postnatal services; the norm was to share space with the admissions room, where women are admitted right before delivery. Only four HP and 1 PHCC had at least two separate rooms for examination/consultation/admission, in addition to rooms for delivery, postnatal care, utility services/activities, and a room for staff. Laboratory areas to conduct various tests (i.e. blood, urine, and/or stool for pregnancy, HIV, syphilis, blood grouping, hemoglobin, proteinuria, etc.) are required at PHCC level or higher as per MoHP regulations. These were available for all five PHCCs and the District hospital, in addition to three HPs and both private facilities.

Most delivery and ANC rooms appeared “clean” upon visual assessment; in 3 HPs, these spaces were characterized as “not clean” based on dirty examination bed, dusty furniture and dirty floor and walls. Only five facilities had a toilet attached to the delivery room; all remaining had a toilet elsewhere at the facility, but in six instances the toilet was either unclean and/or water was not available (Additional file 2: Table S1 shows this in more detail).

#### Antenatal care services and supplies (Table 2)

All facilities (except for 1 HP without any ANMs) provided routine and referral ANC services. Proteinuria and hemoglobin testing were not consistently done, even when laboratory services were available; when laboratory services were not available, most facilities reported referring clients to the nearest private or public laboratory. Tetanus toxoid vaccination was widely available. Iron/folic acid tablets were out of stock in 14 health facilities (including the District hospital), mainly due to a nationwide supply shortage, and delayed distribution by MoHP. Similarly, strips for testing proteinuria (i.e. pre-eclampsia screening) were unavailable in 2 of 5 PHCCs.

**Table 2:** ANC services and supplies by type of health facility (n)

ANC tests prior to and during consultations	District Hospital (*N* = 1)	PHCC (*N* = 5)	HP (*N* = 16)	Private (*N* = 2)	Total (*N* = 24)
Routinely conducted for all ANC clients^a^					
Weighing clients	1	5	15	2	23
Taking blood pressure	1	5	15	2	23
Urine test for protein	1	5	2	2	10
Blood test for anemia	1	5	2	2	10
Conducting group health education sessions	1	3	11	0	15
Tests and services routinely offered (at least once during ANC)	N = 1	N = 5	N = 16	N = 2	*N* = 23
Blood test for anemia	1	4	2	2	9
Blood test for syphilis	1	3	2	2	8
Blood group	0	4	2	2	8
Test for Rh factor	0	3	2	2	7
Urine test for protein	1	4	2	2	9
Urine test for glucose	1	3	2	2	8
Counseling on danger signs for pregnancy, labor/delivery, PNC	1	5	15	2	23
Counseling to come to birthing center for delivery and to bring their ANC card	1	5	15	2	23
Counseling about family planning	1	3	14	2	20
Counseling about HIV/AIDS	0	1	11	2	14
Testing for HIV/AIDS	1	1	5	2	9
Tetanus Toxoid Vaccinations available at the ANC					
Yes, all days ANC available	0	0	3	1	4
Yes, but not all days (only designated days in a month)	1	5	13	1	20
Infection control items in ANC room ^a^					
Soap and running water	1	4	11	2	18
Hand disinfectant (Alcohol hand rub)	0	1	7	0	8
Sharps container	1	5	15	2	23
Essential supplies for basic ANC^a^					
Blood pressure apparatus	1	5	16	2	24
Stethoscope	1	5	16	2	24
Fetoscope	1	5	16	2	24
Adult weighing scale	1	4	15	2	22
Iron and/or folic acid tablets	0	1	8	1	10
Mebendazole/Albendazole tablets	1	3	16	1	21
Urine test strip for protein	1	3	1	2	7

^a^Observed or reported AND functioning

#### Delivery care services, supplies and infrastructure (Table 3)

Overall, 16 of the 24 health facilities had all items needed for infection control with both the private facilities having all the items while the District hospital did not have the disinfecting solution at the time of the study. In contrast, the District hospital had all the other elements to support quality whereas both the private health facilities lacked the physical copy of essential guidelines on managing normal labor and birth and emergency obstetric care. Only 9 facilities stocked injectable anticonvulsants and antibiotics for eclampsia and sepsis, respectively, with health posts being particularly understocked (4 of 16).

**Table 3:**
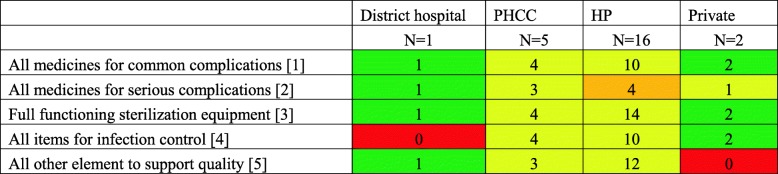
Facilities with available supplies and medicines to delivery quality services by health facility type (n)

Cells are colored according to the number of facilities within each type of health facility having the required supplies and medicines with red indicating availability in none of the facility, yellow indicating availability in half or more than half the facilities, orange indicating availability in less than half the facilities, and green indicating availability in all the facilities

^1^ Needle and syringe, IV solution with infusion set (IV cannula), injectable oxytocin, and perineal/vaginal/cervical repair set located in the pharmacy or delivery room

^2^ Injectable anticonvulsants (magnesium sulfate) and antibiotic (ampicillin or gentamycin) in delivery room or pharmacy

^3^ A working indicator to indicate when sterilization is complete for either the electric or non-electric autoclave with heat source OR a non-electric pot with cover with heat source

^4^ Soap, water, disinfecting solution, puncture proof container, and clean& sterile gloves

^5^ Guidelines, blank partographs, and provider on site or on call 24 h a day

#### BEmONC Signal Functions

**Fig. 1:**
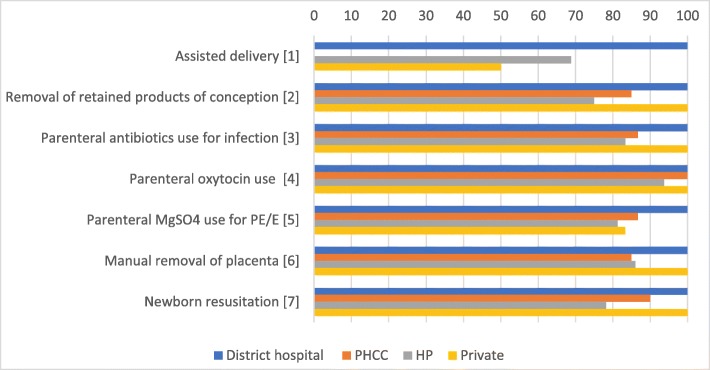
Availability of medications and supplies to perform BEmONC signal functions by health facility type (N = 24). ^1^ Percent of facilities with forceps or ventouse (Vacuum extractor manual or electrical). ^2^Mean percentage score for functioning kit for manual vacuum aspiration or dilation and curettage kit, injectable oxytocin, syringes and needles, and IV solution (ringer’s lactate, dextrose 5% in normal saline (D5NS) or normal saline (NS) infusion). ^3^ Mean percentage score of injectable ampicillin or gentamycin, syringes and needles, and ringer’s lactate, D5NS or NS infusion. ^4^ Mean percentage score of injectable oxytocin, syringes and needles, and ringer’s lactate, D5NS or NS infusion. ^5^ Mean percentage score of injectable magnesium sulfate, syringes and needles, and ringer’s lactate, D5NS or NS infusion. ^6^ Mean percentage score of injectable ampicillin, injectable oxytocin, syringes and needles, and ringer’s lactate, D5NS or NS infusion. ^7^ Mean percentage score of bag and mask (infant size), and resuscitation table for newborn

Four facilities (3 HPs and District Hospital) reported having ever performed the six BEmONC signal functions queried (removal of retained products was not explored), and none reported conducting all six functions within the prior 3 months (data not shown). Mean scores for availability of medications/supplies required for all seven BEmONC signal functions, overall and by facility type are shown in Fig. 1. While the District hospital had capacity to perform all six signal functions and nearly all facilities were equipped to provide oxytocics, less than two-thirds had injectable ampicillin or gentamycin, and no PHCC level facility had manual or electrical vacuum extractors for assisted vaginal delivery; 10 birthing centers lacked injectable magnesium sulfate. The availability of the medicines and supplies to perform the BEmONC signal functions are presented in detail in Additional file 3: Table S2.

#### Newborn Care

The MoHP-indicated list of supplies for quality newborn care was either fully or largely met at the District hospital and the private facilities. However, only 9 facilities maintained a functioning heat source for preterm infants, and only half had oxygen available at the time of assessment. Functioning oral thermometers, suction devices (foot, electric, or DeLee), resuscitation table, and bag/mask were missing from a small number of HPs and PHCCs (Additional file 3: Table S2).

#### Human resources

While the District hospital met MoHP staffing criteria (OB/GYN, ANM/Senior ANM), only 1 of the PHCCs had the requisite staff nurse posted at the time of the audit. Twelve of the sixteen HP level facilities had the required number of ANM/Senior ANM position actually filled while only one of the private facilities had this position filled. At facilities that were not fully staffed, in-charges reported extensive time periods since vacant posts were previously filled (median 48 weeks, range 8 to 520 weeks) and one HP level facility had no ANMs in the prior year.

### Health Worker Assessment and Knowledge

#### Health worker background characteristics, education and training/supervision

We interviewed 63 health workers across the 16 HPs (*n* = 33), 5 PHCCs (*n* = 13), 1 District hospital (*n* = 11) and 2 private clinics (*n* = 6). The majority of health workers (*n* = 54, 85.7%) were ANMs or Senior ANMs, a level which requires grade 10 level education and 18 months training (24 months for Senior certification) (Table 4). At the District hospital maternity ward, one doctor (MBBS, OB/GYN diploma) worked in conjunction with 7 staff nurses (requires grade 10 education and 3 years nursing training), and 4 ANMs. While the MoHP aims to provide an additional two months of SBA training to staff in all birthing centers [18], only half of providers responsible for delivery care in Sarlahi birthing centers had received this training (Table 4). The proportion of staff who reported receiving pre-service or in-service training for ANC, delivery, and newborn care in the past 3 years was 83, 74.6, and 65.1% respectively. However, the majority of District hospital and private clinic staff reported not receiving the refresher trainings. A quarter of the health workers reported never having received supervision or technical support; of these, about 75% were new staff who started working from 2015 onwards. When asked to suggest what could be improved in their working situation in order to support good quality of care, 81% of health workers reported the need for more supplies/drugs (all heath facilities), followed by 57% requesting better facility infrastructure (mostly PHCC and HP level staff), and 30% reporting the need for more knowledge/updates/trainings. The details of the training and work experience are shown in Additional file 4: Tables S3A and S3B by type of health facility and SPA training respectively.

**Table 4:** Distribution of health worker characteristics, training and education by type of health facility

Characteristics	District Hospital (%) N = 11	PHCC (%) N = 13	HP (%) N = 33	Private (%) N = 6	Total (%) *N* = 63
Health Worker Cadre					
Sr. ANM/ANM	27.3	92.3	100	100	85.7
Sr. Staff Nurse/Nurse	63.6	7.7	0	0	12.7
Medical Doctor	9.1	0	0	0	1.6
Age of respondent					
18–25 years	9.1	23.1	39.4	16.7	28.6
26–30 years	54.5	23.1	24.2	16.7	28.6
31–35 years	9.1	15.4	18.2	0	14.3
36–40 years	18.2	15.4	12.1	50	17.5
> 40 years	9.1	23.1	6.1	16.7	11.1
*Median (years)*	*27 years*	*34 years*	*28 years*	*37.5 years*	*29 years*
Highest Professional/Technical/Medical Qualification*					
Bachelor of Medicine and Bachelor of Surgery (MBBS) with OB/GYN diploma	9.1	0	0	0	1.6
BSc Nursing/ Bachelor in Nursing/Bachelor in Health Education	27.3	15.4	3.0	0	9.5
Proficiency Certificate Level (PCL) Nurse	36.4	23.1	18.2	0	20.6
Senior ANM training	0	15.4	0	0	3.2
ANM training	27.3	46.2	75.8	100	63.5
MCHW training	0	0	3.0	0	1.6
Completion of highest level of training year					
Before 2000	9.1	15.4	3.0	33.3	9.5
2000–2005	0	15.4	18.2	33.3	15.9
2006–2010	18.2	23.1	36.4	16.7	28.6
2011–2015	72.7	46.2	42.4	16.7	46.0
*Median year*	*Year 2013*	*Year 2010*	*Year 2010*	*Year 2002*	*Year 2010*
SBA additional training					
Yes	36.4	69.2	51.5	33.3	50.8
No	63.6	30.8	48.5	66.7	49.2

*Fishers exact test *p*-value < 0.05

#### Maternal and newborn health knowledge

**Fig. 2:**
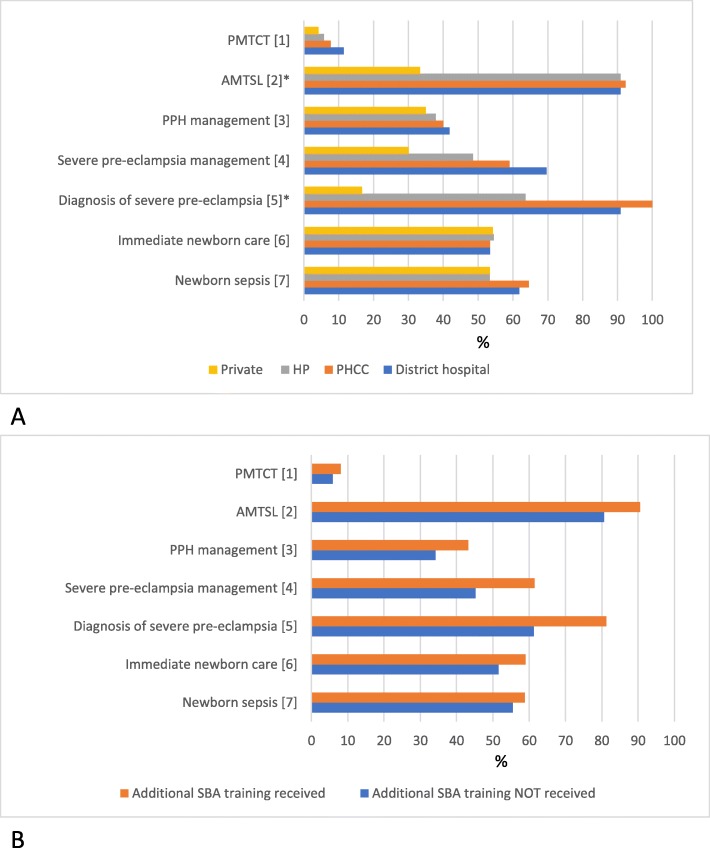
**a.** Percent distribution of maternal and newborn health knowledge among health workers by facility type (N = 63). **b.** Distribution of maternal and newborn health knowledge by health workers with and without SBA training. ^1^ Mean percentage score of knowledge on actions for the prevention of mother-to-child transmission of HIV (PMTCT) during labor and delivery. ^2^ Correct knowledge on all three key steps of active management of third stage of labor (AMTSL) namely administration of uterotonic immediately/within 1 min of delivery, controlled cord traction and uterine massage. ^3^ Mean percentage score of knowledge on actions appropriate for heavy bleeding postpartum from atonic/uncontracted uterus. ^4^ Mean percentage score of knowledge on actions most appropriate in managing woman with severe pre-eclampsia at term. ^5^ Correct diagnosis of severe pre-eclampsia on the case scenario. ^6^ Mean percentage score of knowledge on immediate newborn care after birth and within the first hour baby delivered with no complication. ^7^ Mean percentage score of knowledge on five signs and symptoms of newborn infection (sepsis) namely poor/no breastfeeding, hypo/hyperthermia, restlessness/irritability, and breathing difficulties. * Fishers exact test *p*-value < 0.05

Figure 2a and b illustrate the distribution of maternal (5 components) and newborn (2 components) health knowledge among the health workers by type of facility and SBA training status respectively with detailed breakdown in the tables in Additional file 5 and Additional file 6. Overall, the majority of health workers had complete knowledge on active management of third stage of labor (AMTSL) and to some extent the diagnosis of severe pre-eclampsia (but not management of pre-eclampsia) with better knowledge among the public sector health workers. Knowledge of components of prevention of mother to child transmission (PMTCT) of HIV was generally poor. Health workers’ knowledge on other topics of maternal and newborn health was moderate. The SBA trained health workers had higher knowledge across all topics, but none of these differences were significant.

## Discussion

We identified some critical gaps in facility readiness and health worker preparedness to provide quality ANC, labor/delivery and immediate newborn care services. Due to these gaps, the majority of the birthing centers did not meet the requirements set by NSMP guidelines. A consistent barrier to readiness to provide quality services was the lack of medicines and supplies needed for vital ANC, delivery, and newborn care services. The majority of the health workers at all facility levels agreed that more drugs and supplies are needed for improved service quality. The nationwide supply shortage of iron/folic acid tablets for more than a year affected stocks in majority of the facilities, which is in contrast to the finding from the 2015 Nepal Health Facility Survey (NHFS) that showed 90% facilities having iron/folic acid tablets [30]. Lack of anticonvulsants (e.g. magnesium sulphate) to manage severe pre-eclampsia/eclampsia, which is the second major cause of maternal deaths [31], and lack of vacuum extractor to conduct assisted deliveries in all of the PHCCs could also adversely affect the ability of skilled staff to provide quality care, thus increasing the risk of maternal and perinatal mortality and morbidity. On the other hand, distribution of oxytocics appeared universal, which is reassuring, given that hemorrhage is the most common cause of maternal mortality [31]. Such accomplishments must be extended to a broader set of supplies, and ensured across all health facilities in order to reduce delay in provision of lifesaving interventions.

The 2015 NHFS found that centers designated as BEmONC-capable did not necessarily have this capacity due to gaps in drugs and equipment; lack of magnesium sulfate, injectable antibiotics, and MVA/D&C kits were particularly glaring [30]. We have previously reported that in this community women initially seek care for maternal and newborn illnesses from informal providers such as local village doctors, traditional birth attendants and traditional healers [32]. In instances where care was sought at a health facility, adequate care was sometimes further delayed due to shortages of drugs and supplies resulting in referral or the need for the family or patient to purchase the supplies and medicines [32]. Our findings in this study are consistent with these earlier results, confirming that many facilities do not have the basic commodities required to provide essential delivery and newborn services.

Birthing centers established without sufficient physical infrastructure (i.e. rooms and laboratories as per NSMP guidelines) result in sharing beds/space across labor and postnatal services, and forces referral to external labs for even basic testing services. Thus, in many facilities, lab tests which are an integral part of ANC, were not done either because they did not have the facilities or did not have the testing supplies. Pregnant women are more likely to not have any laboratory tests done due to distance to nearest referral lab or financial constraint (personal communication-health workers). This can possibly lead to delayed diagnosis or non-diagnosis of a potential pregnancy complication (such as diagnosis of protein in the urine to diagnose severe pre-eclampsia, or blood glucose level tests to diagnose gestational diabetes etc.), resulting in inability to take preventive measures or receive treatment in a timely manner. An assessment of service readiness of health facilities in ten countries, including Nepal, found that only 2% of facilities surveyed between 2007 and 2015 had eight diagnostic tests defined as essential for basic service readiness by the WHO, including those necessary for antenatal care, such as urine dipsticks for protein and glucose, syphilis rapid diagnostic tests, and HIV diagnostic capacity [33]. In many LMICs, the lack of laboratory facilities and diagnostic equipment remains a barrier to effective patient assessment and diagnosis.

Long-standing staffing gaps or sub-optimal distribution of human resource assets in many PHCC and HP level facilities is a result of insufficient SBA training programs, ineffective deployment, and poor worker retention, and reflects typical health system bottlenecks in quality care in many developing countries like Nepal [34]. Skilled health worker shortages remain a major barrier to facility readiness to provide BEmONC services in many countries in the region [35, 36]. Regular supervision and technical support, which are important to create an enabling work environment, need to be emphasized in birthing centers with newer recruits who are most likely to be missed [37]. The prior national birthing center assessment also showed overall about 30% of health workers reporting never being supervised, with the Terai region being slightly higher (36.5%) [24]. Health worker knowledge on the topic of PMTCT was low, perhaps due to the lack of emphasis in training programs, as HIV risk is low in the general population of Nepal and training on PMTCT is centered on the staff of HIV Counseling and Testing sites that operate separately from the birthing centers [38]. Knowledge of AMTSL, which is an essential step to prevent postpartum haemorrhage in normal vaginal delivery [39], was high among the public sector staff but significantly lower among the private sector staff; this may reflect the value of annual in-service training provided to the public sector birthing center health workers in the PHCCs and HPs on maternal and newborn care by the District Public Health Office. In addition to in-service training, health-care providers also need good working conditions, clinical support and opportunities to learn and grow so as to remain motivated and committed to providing high-quality care [40, 41].

Our evaluation showed that the private birthing centers had high standards of care in terms of cleanliness, infrastructure, infection control, medicines/supplies and human resource, but did not necessarily have high performance in some of the maternal and newborn knowledge indicators. This may in part be due to the exclusion of the private sector in the refresher trainings and SBA trainings. In Sarlahi district, pregnant women who deliver in either one of the private clinics are eligible to receive the transportation cost reimbursement from MoHP but the exclusion of the private sector staff in trainings is a service gap that could be strengthened through public-private partnership (PPP) efforts [42]. One example of PPP is the state led Chiranjeevi Yojana (CY) program of Gujarat state in India implemented to full scale in 2007 that used explicit performance-based subsidies to motivate private sector obstetricians to provide institutional delivery services at a defined level of quality and at an affordable cost to disadvantaged women [43]. Similar direct reimbursement to the health providers such as nurse/ANMs in accredited private birthing centers and clinics (preferably with an obstetrician) can be explored for situations when the public sector facilities capacity to provide emergency obstetric care is low [44].

Compared to the HPs/PHCCs, the district hospital had better service readiness, especially in terms of medicines and supplies, basic infrastructure and human resources. Similar findings of better service readiness in hospitals compared to primary care facilities is evident in other settings as well [33, 45]. Studies have shown that healthcare utilization patterns, retention in care and people’s decision to bypass facilities is due to the patient’s perception of quality [46, 47]. In Nepal [48], India [15, 49] and some sub-Saharan Africa countries [50, 51], bypassing the nearest public health facility for a higher level facility or private facility due to at least one quality concern is evident. Since birthing centers at the HP level, especially in rural areas, are the first line of care, these facilities should be given increased priority for equipment to prepare them to provide the basic services which will hopefully and prevent bypassing of them for basic maternal and newborn care.

The MoHP and key stakeholders should establish standard regulations and routinely monitor readiness by type of health facility, identify gaps and areas for improvement, and intervene accordingly at the district level. Currently, MoHP monitors the Safe Motherhood Program through service coverage indicators collected monthly at the facility level; such indicators include the % of women who received a 180 day supply of iron/folic acid during pregnancy, % institutional deliveries (out of population-based estimate of expected livebirths), ANC coverage (4 visits, at least one), etc. [52]. However, monitoring of service coverage and aiming for high levels of service coverage without regard to quality of service will not help achieve the ambitious maternal, newborn and child health goals. In most low-income countries, ANC quality has lagged behind ANC coverage, where 86·6% (83·4–89·7) of women accessed care but only 53·8% (44·3–63·3) reported receiving the three services (blood pressure monitoring and urine and blood testing) [53]. Similarly, the Commission on HQHS analysis showed that out of 16 LMICs, adherence to WHO guidelines in improving ANC, family planning and sick child care services was low, with Nepal being one of the low performing countries [15]. Nepal has made a commitment towards building a high-quality health system, but progress has been slow and steps have been proposed that go beyond vertical programs in the context of the new federal system of governance [54]. The need to strengthen the procurement and distribution chain for basic drugs and equipment and the need to improve skills of providers to ensure at least a minimum coverage of BEmONC is available cannot be overemphasized and should be closely monitored by the local and provincial authorities who are now responsible for procurement in the federal system. Further research on the quality of care in the public health facilities provided, and the challenges remaining after the transition from the central governance to local/province level governance is warranted.

Strengths of our study include utilizing existing tools allowing comparison at the national level on key indicators, universal sampling of all birthing centers in the district, and interviews with all the health workers employed in the birthing centers during the study period, thus providing a complete picture of health worker knowledge in this district, which is typical of much of rural Nepal. Limitations include the use of self-reported information on the performance of BEmONC signal functions and the lack of observation-based measures of obstetric care, which is considered the gold standard for measuring process quality but is resource-intensive to implement. Other important dimensions of quality, including communication among providers, both within and between facilities, the quality of documentation, and the strengths and weaknesses of referral systems were not directly assessed. Our more narrow focus, however, was still able to identify several inputs needed to provide high-quality obstetric care were not in place and provided actionable data on health facility readiness to health officials. The Commission on HQHS argues that quality measurements have predominantly focused on inputs and that they provide a insight into quality of care and proposes accountability and action as the guiding purposes of quality improvement [15]. Our study identified gaps in the birthing center readiness to provide the basic services and the findings were shared with district public health office key staff (such as district public health officer, the public health nurse) who are accountable at the district level to ensuring the gaps are resolved.

We assessed health worker knowledge about key aspects of maternal and newborn health. Knowledge (assessed through test and case scenarios) and performance are not very closely correlated [24, 27, 28, 55, 56]: health workers who provide appropriate care may still have difficulty answering questions correctly, and those who answer questions correctly may not provide appropriate care. However, most health facility surveys only collect data on recent health worker training; health worker competence is an essential precondition for high quality consultations, so data on health worker knowledge is a useful complement to training data. We also did not capture the humanization aspect of QoC, which would be important to understand as disrespect and abuse are distressingly common and might dissuade women from seeking care (8,37).

## Conclusion

In Nepal, financial incentives for women, performance-based financing for providers and facilities, and removal of user fees have increased facility births and ANC visits. Such rapid increase in utilization is likely to place a considerable burden on the facilities and may compromise quality [57, 58]. This study shows that the gaps in quality of essential maternal and newborn care remains a major challenge at all levels and differ by type of facility in rural Nepal. To reduce the burden of maternal and newborn deaths and to achieve the SDGs, we need to overcome both the “coverage gap” and the “quality gap” [12, 59]. Routine and robust monitoring of health facilities to ensure readiness is an important first step towards improving quality [60]. As the government transitions into the federal system of government, it is essential for each provincial government to monitor and assess service quality gaps, since it is evident that gaps vary by facility type and so improvement efforts should be adapted for local context and monitored. In addition to periodic national birthing center assessments, focused maternal and newborn QoC assessments at the district level through regular monitoring, audits, supervisions and refresher trainings are also required.

## Supplementary information

**Additional file 1.** Health Facility Audit Tool and Health Worker Knowledge Interview Guide. This additional file contains the health facility audit tool and the interview guide to assess health worker knowledge on maternal and newborn care.

**Additional file 2: **Labor/delivery room and ANC examination room setting. This additional file includes **Table S1.** which shows the results on the setting of the labor/delivery room and ANC examination room and basic infrastructure in those rooms.

**Additional file 3: **Capacity to perform BEmONC signal functions and immediate newborn care. This additional file includes **Table S2.**, which shows the detail breakdown of the availability of the medicines and supplies to perform each of the seven BEmONC signal functions as well as the availability of medicines/supplies to deal with common or serious complications and immediate newborn care.

**Additional file 4: **Health worker training and work experience. This additional file shows the detailed breakdown on the type and years of training the health workers have received along with their number of years of experience working in ANC, delivery care and newborn care. **Table S3A.** and **S3B.** displays the results on training and work experience of the health workers by health facility type and SBA training respectively.

**Additional file 5: **Health worker knowledge on maternal health. This additional file shows the detailed breakdown on health worker knowledge on various topics of maternal health by type of health facility (**Table S4A.**) and by SBA training (**Table S4B**).

**Additional file 6: **Health worker knowledge on newborn health. This additional file shows the detailed breakdown on health worker knowledge on various topics of newborn care by type of health facility (**Table S5A.**) and by SBA training (**Table S5B**).

## Data Availability

De-identified data underlying these findings are available in the JHU Data Archive: 10.7281/T1/2K70CX

## Abbreviations

AMTSL: Active Management of Third Stage of Labor
ANC: Antenatal care
BEmONC: Basic Emergency Obstetric and Newborn Care
CEmONC: Comprehensive Emergency Obstetric and Newborn Care
HP: Health Post
HQHS: High-quality Health Systems
LMIC: Low-middle Income Country
MDG: Millennium Development Goals
MMR: Maternal Mortality Ratio
MoHP: Ministry of Health and Population
NHFS: Nepal Health Facility Survey
NMR: Neonatal Mortality Rate
NSMP: Nepal Safe Motherhood Program
PHCC: Primary Health Care Center
PMTCT: Prevention of Mother to Child Transmission
PPP: Public-Private Partnership
QoC: Quality of Care
REDCap: Research Electronic Data Capture
SBA: Skilled Birth Attendant
SDG: Sustainable Development Goals
