# Supplementary material for: Assessment of facility and health worker readiness to provide quality antenatal, intrapartum and postpartum care in rural Southern Nepal
Source: BMC Health Serv Res. 2020 Jan 6;20:16. doi: 10.1186/s12913-019-4871-x (PMC6945781; doi:10.1186/s12913-019-4871-x)
Supplement: Supplementary file 1 — Additional file 1. Health Facility Audit Tool and Health Worker Knowledge Interview Guide. This additional file contains the health facility audit tool and the interview guide to assess health worker knowledge on maternal and newborn care. [file 12913_2019_4871_MOESM1_ESM.pdf]

Additional File 1: Health Facility Audit Tool and Health Worker Knowledge Interview Guide

BIRTHING CENTRE AUDIT TOOL

Week [ ][ ][ ] Date [ ][ ]-[ ][ ]-[ ][ ]
DD MM YY

VDC [ ][ ] Ward [ ] Type of Health Facility [ ]

1=PHCC
2= HP
3=Private clinic
4 = Hospital

Health facility code [ ][ ]

Find the facility director or in-charge.

Give consent form to facility director or in-charge.

Ask facility director/in-charge Do I have your agreement to proceed?

F1: Was permission received from director/in-charge to participate in study?

☐ Yes, permission was given to ask questions about the facility → go to Q.1

☐ No, the in-charge declined to offer information about the facility → STOP

Section 1: Infrastructure				
Read questions aloud to Facility Director/In-charge				
	Code			Go to
Q101: How long does it take to walk to the nearest motorable road (big enough for a four wheel vehicle) (in minutes)? (write '00' if linked to the birthing centre)	☐☐			
Q102: How is the transportation status to reach the District hospital from the Birthing Centre?				
Motorable road throughout	1			
Some section Motorable road and some section on foot/two-wheeler	2			
	☐☐☐			
Q103: Total time to drive to the District Hospital from the Birthing Centre (in minutes)?				
Q104: Which year was this birthing centre established (Roman calendar)?				
	☐☐☐☐			
Q105: Is this birthing centre owned or rented?				
Its own	1			
Rented	2			
Other (specify)______________	3			
Q106: Does the birthing centre have the following:	Yes Separate		Yes, but not separat e	
a) Examination/consultation/admission room (at least 2)	No			
b) Delivery room	1	0	2	
c) Postnatal room	1	0	2	
d)Laboratory	1	0	2	
e) Pharmacy	1	0	2	
f) Reception and waiting area	1	0	2	
g) Area for family members (sleeping, eating etc.)	1	0	2	
h) Utility room	1	0	2	
i) Staff room (for night service)	1	0	2	
	1	0	2	
Q107: Does this facility have a working phone to call outside that is available at all times client services are offered? (clarify that if 24 hour services are offered, this refers to 24 hour availability)			2	
Yes, onsite or within 5 mins walk	1			
Yes, within 5 min, not onsite	2			
Only pay phone or personal cell phone	3			
No	0			

Q108: Does this facility have a functional ambulance or other vehicle on-site for emergency transportation of clients? IF yes, ask if the vehicle is functioning and if there is fuel available. (Accept reported response.)	
Yes, functioning with fuel	1
Yes, not functioning or no fuel	2
No	0
Q109: What is the usual mode of transport the people in this VDC/Municipality use to bring the woman in labor at this birthing centre?	
Ambulance	1
Bullock cart	2
Rickshaw	3
Rented motor vehicle	4
Own motor vehicle	5
Brought manually in the basket/stretcher	6
Q110: What is done to dispose of the placenta (eg: placenta pit) ?	
End of Section 1	

Section 2: Labor & Delivery Inventory

Ask to speak with the head of labor & delivery unit (this may be director/in-charge if no head of unit)

Question	Yes	No	DK	Go to
Q201: Does this facility provide 24 hour coverage for delivery services?	1	0	98	No→Q203a
Q202: Is a person skilled in conducting deliveries present at the facility or on call 24 hours a day, including weekends, to provide delivery care?	Code			Go to
Yes, present, schedule observed	1			
Yes, present, schedule reported, not seen	2			
Yes, on-call schedule observed	3			
Yes, on-call, schedule reported, not seen	4			
No	0			

Read aloud: Now I am going to ask you about medical interventions for management of complications during labor or delivery. For each intervention, please tell me if this is ever provided at this facility, and if yes, if it has been conducted in this facility within the past 3 months.

Question	Yes	No	DK	Go to
Q203a: Does this facility ever provide parenteral oxytocic drugs for pregnancy-related hemorrhage	1	0	98	No→204a
Q203b: In the past 3 months	1	0	98	
Q204a: Does this facility ever provide parenteral anticonvulsants for pregnancy-related hypertension	1	0	98	No→205a
Q204b: In the past 3 months	1	0	98	
Q205a: Does this facility ever provide parenteral antibiotics for pregnancy-related infections	1	0	98	No→206a
Q205b: In the past 3 months	1	0	98	
Q206a: Does this facility ever perform manual removal of placenta	1	0	98	No→207a
Q206b: In the past 3 months	1	0	98	
Q207a: Does this facility ever perform newborn resuscitation	1	0	98	No→208a
Q207b: In the past 3 months	1	0	98	

Q208a: Does this facility ever perform assisted deliveries—that is, use ventouse (vacuum extractor)	1	0		No→209
Q208b: In the past 3 months	1	0	98	

Read aloud: Now I want to ask you about how this facility handles contaminated reusable equipment after completing a delivery. If the unit processes some equipment and sends other equipment elsewhere, indicate the procedure for equipment processed in this service delivery unit. If vaginal deliveries are conducted in a different room than caesarean section deliveries, assess the processing equipment for vaginal deliveries.

Q209: After completing a delivery, what procedures do health workers follow for initial handling of contaminated equipment (such as speculums, scalpel handles, etc.) that will be reused another time? [Note: multiple response possible – upto three options]	Code	Go to
Disinfectant, then soap & water scrub	1	
Soap & water scrub, then disinfectant soak	2	
Soap & water brush scrub only	3	
Disinfectant soak, not scrubbed	4	
Soap & water, not brush scrubbed	5	
Nothing is done	6	
Other (specify)_______	7	
Don't know	98	

Q210: Besides decontaminating and cleaning, what is the final process most commonly used for disinfecting or sterilizing medical equipment (such as surgical instruments) before they are reused? If different methods are used for different types of equipment, indicate the method(s) used for metal equipment such as speculums or forceps.

Dry-heat sterilization	1
Autoclaving	2
Steam sterilization	3
Boiling	4
Chemical method	5
Nothing is done	6
Other (specify)_______	7
Don't know	98

There are no more questions for the head of labor & delivery unit/director. explain that for the next section, you will need to walk around and look at the delivery service area. They can now choose to accompany you for the rest of the assessment or attend to other business. If they do not accompany you, ask if a health worker involved in delivery care can help you with the next part of the assessment.

Ask to see the room where normal deliveries are conducted.		
Q211: Describe the setting of the delivery room	Code	Go to
Private room with visual and auditory privacy	1	
Non-private room with visual and auditory privacy	2	
Visual privacy only	3	
No privacy	4	
Q211a. How many rooms separated for delivery care?	☐	
Q212: Describe the conditions in the delivery room:		
Clean	1	
Dirty	2	
Don't Know	98	
Q212a: Is there a toilet for client use? near the delivery room		
Yes, attached to the delivery room	1	
Yes, but not attached to the delivery room	2	
No	0	→213
Q212b: Is the toilet functioning (clean, water availability/bucket)?		
Yes	1	
No	0	
Don't Know	98	
Q213: Is there electricity?		
Yes	1	
No	0	
Don't Know	98	
Q214: Is there backup electricity supply (inverter, solar, generator etc.) during load shedding?		
Yes	1	
No	0	
Don't Know	98	

Note the availability and condition of the following supplies, equipment and medications needed for delivery services. Items may be in delivery room or an adjacent room. If you do not see an item, ask the health worker helping you to show you the item.

SUPPLIES AND EQUIPMENT IN DELIVERY ROOM	Observed	Reported not seen	Not available	Don't know	Go to
Q215: Clean and sterile gloves	1	2	3	98	
Q216: Puncture proof container	1	2	3	98	
Q217: Use of 5% Chlorine	1	2	3	98	
Q218: Stainless steel drums for gloves/instruments	1	2	3	98	
Q219: Containers to collect waste	1	2	3	98	
Q220: Soap for handwashing	1	2	3	98	
Q221: Water for handwashing	1	2	3	98	No/DK→222a

Question	
Q221a: How is water being made available for use in the delivery service area today?	Code
Piped	1
Bucket with tap	2
Bucket or basin	3
Not available today	0

	Observed	Reported not seen	Not available	Don't know	Go to
Q222a: Syringes	1	2	3	98	
Q222b Needles	1	2	3	98	
Q223: Sterile scissors or blade	1	2	3	98	
Q224: Sterile disposable cord ties or clamps	1	2	3	98	

Q225: Chlorhexidine (CHX) available	1	2	3	98			
Q226: Towel or blanket to wrap baby	1	2	3	98			
Q227: Blank partographs or blank maternity booklets with partograph included	1	2	3	98			
	Availability (a) (Skip section b if not available or Don't know)				Functioning (b)		
	Observed	Reported not seen	Not available	Don't know	Yes	No	DK
Q228: Incubator	1	2	3	98	1	2	98
Q229: Other source of heat for premature infant (If yes, Specify) ______	1	2	3	98	1	2	98
Q230: Bag and mask (infant size) for resuscitation	1	2	3	98	1	2	98
Q231: Foot suction or electric suction or Delees for mucus extraction	1	2	3	98	1	2	98
Q232: Suction apparatus for use with catheter	1	2	3	98	1	2	98
Q.233: Cathether foles or rubber for retained placenta	1	2	3	98	1	2	98
Q234: Resuscitation table for baby	1	2	3	98	1	2	98
Q235: Ventouse (vacuum extractor - manual or electrical) for obstructed labor	1	2	3	98	1	2	98

Q236: Manual vacuum aspirator (MVA)	1	2	3	98	1	2	98
Q237: Dilatation and curettage (D&C) kit	1	2	3	98	1	2	98
		Repo	Not				
Q238: GENERAL EQUIPMENT	Obser	rted,	Avail				
AND INSTRUMENTS	ved	Not	able	DK			
A. Instrument Trolley (at least 1)	1	seen	3	98			
B. Stethoscope	1	2	3	98			
C. BP measuring apparatus	1	2	3	98			
D. Fetoscope	1	2	3	98			
E. An Oral thermometer	1	2	3	98			
F. A Drum for gloves	1	2	3	98			
G. A Cheattle Forceps with Jar	1	2	3	98			
H. A Measuring tape	1	2	3	98			
J. An Oxygen cylinder	1	2	3	98			
K. A Portable light	1	2	3	98			
L. Emergency light	1	2	3	98			
M. Wall clock	1	2	3	98			
N. IV sets (can be attached to	1	2	3	98			
bed)	1	2	3	98			
O. Electronic suction Equipment	1	2	3	98			
P. Episiotomy set	1	2	3	98			
Q. Perinea /Vaginal / cervical		2					
repair set							
	1		3	98			
Q239: FURNITURE:	1	2	3	98			
	1	2	3	98			
A. An examining Bed/Table;	1	2	3	98			
B. Delivery Tables/beds		2					
C. Steps Foot	1		3	98			
D. At least 2 mattresses with	1	2	3	98			
water proof covers	1	2	3	98			
E. At least 2 pillows with water	1	2	3	98			
proof covers	1	2	3	98			
F. Screen	1	2	3	98			
G. Revolving stool	1	2	3	98			
H. Cupboard		2					
I. Table							
J. At least 2 Chairs							

If medications are packaged together in a combo-pak, circle yes for each individual medication in the pack

Q240: MEDICATIONS in delivery room	Observed ≥1 valid dose	Reported not seen	Not available	Don't know	
A) Intravenous solutions: either Ringers lactate, D5NS, or NS infusion	1	2	3	98	
B) Calcium Gluconate 10ml	1	2	3	98	
C) Injectable oxytocin 1 IU/mL	1	2	3	98	
D) 25% dextrose (2 amp)	1	2	3	98	
E) Injectable magnesium sulfate 2cc/1gm	1	2	3	98	
F) amoxicillin or injectable ampicillin	1	2	3	98	
G) Injectable gentamicin	1	2	3	98	
H) Hypertensive drug (nefidipine 5/10 mg or other)	1	2	3	98	
I) IV Cannula (1 set)	1	2	3	98	
Guidelines may be printed or handmade.					
GUIDELINES/ PROTOCOLS IN DELIVERY ROOM	Observed	Reported not seen	Not available	Don't know	
Q250: Guidelines for care/managing normal labor and birth	1	2	3	98	
Q251: Guidelines for emergency obstetric care	1	2	3	98	
Ask the health worker to show you where delivery equipment is sterilized.					
	Availability (a)				Functioning (b)

	(Skip section b if not available or Don’t know)						
EQUIPMENT USED FOR STERILIZATION	Observed	Reported not seen	Not available	Don’t know	Yes	No	DK
Q253: Electric autoclave (Pressure and Wet Heat)	1	2	3	98	1	2	98
Q254: Non-electric autoclave (Pressure and Wet Heat)	1	2	3	98	1	2	98
	1	2	3	98	1	2	98
Q255:Electric boiler or steamer (no pressure)	1	2	3	98	1	2	98
Q256: Non-electric pot with cover (for steam/boil)	1	2	3	98	1	2	98
Q257: Heat source for non-electric equipment	1	2	3	98	1	2	98
Q258: TST Indicator strips or other item that indicates when sterilization is complete.	1	2	3	98	1	2	98
Q259: Does this facility practice Kangaroo Mother Care (Maya ko Angalo) for low birth weight babies?<br><br>Yes<br>No	Code<br><br>1<br>0						
End of Section 2							

Section 3: Antenatal Care Inventory

Ask to speak with the head of antenatal care unit (this may be director/in-charge if no head of unit)

Question	Yes	No			Go to
Q300: Does this facility offer routine antenatal services?	1	0			
Q301: Does this facility offer referral antenatal services?	1	0			Q300 is no and Q301 is no→ end section
Q302: Does this facility have a system whereby measurements or procedures for ANC clients are routinely carried out before the consultation?	1	0			No→Q308

Ask to see the place where antenatal clients are seen before they have their medical consultation and indicate which of the following activities are routinely carried out there. Observe if the below activities are being conducted routinely. If not seen ask:

Is [read activity you do not see] routinely conducted for all antenatal care clients?	Observed	Reported not seen	Not available	DK	Go to
Q303: Weighing clients	1	2	3	98	
Q304: Taking blood pressure	1	2	3	98	
Q305: Urine test for protein	1	2	3	98	
Q306: Blood test for anemia	1	2	3	98	
Q307: Conducting group health education sessions	1	2	3	98	

Which of the following activities are performed as part of routine	Yes	No	DK	Refereal only	Go to

services, that is, each client has this test at least once.					
Q308: Blood test for anemia	1	0	98	2	
Q309: Blood test for syphilis	1	0	98	2	
Q310: Blood group	1	0	98	2	
Q311: Test for Rh factor	1	0	98	2	
Q312: Urine test for protein	1	0	98	2	
Q313: Urine test for glucose	1	0	98	2	
Q314: Counseling on danger signs for pregnancy, labor/delivery, PNC	1	0	98	2	
Q. 315: Counseling pregnancy women to come to BC for delivery and to bring their ANC card	1	0	98	2	
Which of the following types of treatment and services are routinely offered to antenatal clients?					
Q316: Counseling about family planning	1	0	98		
Q317: Counseling about HIV/AIDS	1	0	98		
Q318 Testing for HIV/AIDS	1	0	98		
Q319: Is tetanus toxoid vaccination available all days antenatal care services are offered?	Code				Go to
Yes	1				
Not all days	2				

Never offered	3	
Q320: How many days each month are tetanus toxoid vaccinations offered at this facility or by this facility outside in the community? (If never offered, enter 00, don't know enter 98)	☐☐	

There are no more questions for the Head of antenatal care unit/director. explain that for the next section, you will need to walk around and look at the antenatal care examination area. They can now choose to accompany you for the rest of the assessment or attend to other business. If they do not accompany you, ask if a health worker involved in antenatal care can help you with the next part of the assessment.

Ask to see the room where examinations for antenatal clients are conducted.

Q321: Describe the setting of the ANC examination room	Code	Go to
Private room with visual and auditory privacy	1	
Non-private room with visual and auditory privacy	2	
Visual privacy only	3	
No privacy	4	
Q322: Describe the conditions in the ANC examination room		
Clean	1	
Dirty	2	
Don’t Know	98	
Q323: Is there a toilet for client use near the ANC service delivery area	Code	Go to

Yes	1	
No	0	→Q324
Q323a: Is the toilet functioning (clean, water availability/bucket)?		
Yes	1	
No	0	
Don’t know	98	

Note the availability and condition of the following supplies, equipment and medications needed for ANC services. Items may be in the room where ANC examinations take place or an adjacent room. If you do not see an item, ask the health worker helping you to show you the item.

SUPPLIES AND EQUIPMENT IN ANC EXAMINATION ROOM	Observed	Reported not seen	Not available	Don’t know	Remarks
	1	2	3	98	
Q324) Sharps container	1	2	3	98	
	1	2	3	98	
Q325) Alcohol hand rub	1	2	3	98	
Q326) Waste receptacle with lid and plastic liner	1	2	3	98	
Q327) Soap for handwashing	1	2	3	98	

Equipment may be in examination room, an adjacent room, or room where measure is taken.

	Availability (a)<br>(Skip section b if not available or Don’t know)				Functioning (b)		
EQUIPMENT AND TESTING SUPPLIES	Observed	Reported not seen	Not available	Don’t know	Yes	No	DK
Q328) Blood pressure apparatus	1	2	3	98	1	2	98

Q329) Stethoscope	1	2	3	98		1	2	98
Q330) Fetal stethoscope (Fetoscope)	1	2	3	98		1	2	98
Q331) Adult weighing scale	1	2	3	98		1	2	98
	1	2	3	98		1	2	98
	1	2	3	98		1	2	
Q332) Urine test strip for protein								9
Q333) Oral thermometer								8
	1	2	3	98		1	2	98
	1	2	3	98		1	2	9
	1	2	3	98		1	2	8
Q334) HIV rapid test / HIV Determine and Unigold	1	2	3	98		1	2	98
Q335) Measuring tape								98
MEDICATIONS/ VACCINE	Observed	Reported not seen	Not available	Don't know	Go to			
Q336) Iron and/or folic acid	1	2	3	98				
	1	2	3	98				
Q337) Mebendazole/Albendazole tablets	1	2	3	98				
End of Section 3								

Section 4: Human Resource
Ask to speak with the director/in-charge and fill the following:

Q401. Note down the information below as per reported

SL No	Position	A. Number of Post Designated	B. Number of Position Manned	C. If any Position not Manned/Absent, since When? (in weeks)
1	Medical Doctors			
2	Staff Nurses			
3	Health Assistants			
4	AHW / Senior AHW			
5	ANM / Senior ANM			
8	Others Specify			
Total				

Q.402. How many of the health workers are trained to conduct deliveries at the birthing center (ANM require 18 months training to be qualified)?
___________ Number

Q403. How many of the person(s) who is working to handle the deliveries at the birthing centre received the SBA training (additional two months training required by MoHP)?
_______ Number

End of Section 4

Section 5: Health Facility Records/Posters

Ask to speak with the director/in-charge and fill the following:

Q501. Note down the information below as per observation

SL No	Medical records/poster	Observed Yes=1 No=1

1	Anusuchi dosh (Monthly Client Information)	
2	Chlorohexidine use Cord Care Poster	
3	Jeevan Surakshya Flip Chart or Poster (Maternal and Newborn Protection)	
4	Pregnancy danger signs	
5	Newborn danger signs	
6	Family Planning Chart /Poster	
7	Magnesium Sulphate Use Guide	

Comments : _________________

End of Section 5

End of Health Facility Audit

Health Worker Interview and Knowledge Assessment

Week ☐☐☐ Date ☐☐-☐☐-☐☐
DD MM YY

VDC ☐☐ Ward ☐ Type of Health Facility ☐

1=PHCC
2= HP
3=Private clinic
4 = Hospital

Health Facility code ☐☐

Explain to the health worker that his/her name was provided as a knowledgeable maternal and/or neonatal health provider. Validate with the health worker that he/she does provide some maternal and/or neonatal health services in this facility.

1: Ask health worker Have you given your consent to participate in the study?

☐ Yes → go to 2

Read oral consent script to health worker.

2: Ask health worker Do I have your agreement to proceed?

☐ Yes, consent is given → go to 3

☐ No, consent is not given → observation of this health worker must END; if available, approach another health worker for participation.

3. Position of health worker: ☐ 1= Doctor, 2= Staff Nurse, 3= Auxiliary Nurse Midwife

Section 1: Education, Training and Working Conditions			
Read the following questions to the health worker. If health worker doesn't know the year, probe using past events and record your best estimate.			
Question	Code		
EDUCATION AND EXPERIENCE			
Q101: What is your current professional/technical/medical qualification?			
Q102: What year did you graduate (or complete) with this qualification?<br>[roman calendar]	☐☐☐☐		
Q103a: In what year did you start working in this facility?<br>[roman calendar]	☐☐☐☐		
Q103b: In what year did you start working in your current position in this facility? [roman calendar]	☐☐☐☐		
Q104: What is your age?	☐☐		
TRAINING AND SERVICES PROVIDED			
Question	Yes	No	Go to
Q105: In your current position, and as a part of your work for this facility, do you personally provide any antenatal services?	1	0	No→Q108
Q106: How many years in total have you provided such services? Service may have been here or in another facility(Observer: enter 00 if less than 1 year of service)			
Q107a: During the past 3 years have you received any pre- or in-service training on subjects related to antenatal care?	1	0	No→Q108
Q107b: In the past 3 years, did you receive any training on the following topics (read each answer aloud):	1	0	
ANC screening (e.g., blood pressure, urine glucose and protein)	1	0	
Counseling for ANC (e.g., nutrition, FP and newborn care)	1	0	

PMTCT or other HIV/AIDs related	1	0	
Management of pre-eclampsia/eclampsia	1	0	
Other topic related to ANC	1	0	No→Q108
5a) List other topic:			
Q108: In your current position, and as a part of your work for this facility, do you personally provide any delivery services? By that I mean conducting the actual delivery of newborns	1	0	No→Q114
Q109: How many years in total have you provided such services? Service may have been here or in another facility (Observer: enter 00 if less than 1 year of service)			
Question	Code		
Q110: How often do you use a partograph fully to monitor and manage labor (read each answer aloud):			
Never	1		
Rarely	2		
Sometimes	3		
Most of the time	4		
Always	5		
Q111: How often do you use active management of the third stage of labor (AMTSL) during normal vaginal births (read each answer aloud):			
Never	1		
Rarely	2		
Sometimes	3		
Most of the time	4		
Always	5		
Question	Yes	No	Go to
Q112: During the past 3 years have you received any pre- or in-service training on subjects related to delivery care?	1	0	No→Q114
Q113: In the past 3 years, did you receive any training on the following topics (read each answer aloud):	1	0	
Routine care for labor and normal vaginal delivery	1	0	
Use of partograph	1	0	
Active management of third stage of labor (AMTSL)	1	0	

Sterile cord care and appropriate cord care with chlorhexidine	1	0	
Management of sepsis, including use of parenteral antibiotics	1	0	
Administer magnesium sulfate for the treatment of severe pre-eclampsia or eclampsia	1	0	
Management of postpartum hemorrhage	1	0	
Removal of placenta or products of conception? (D&C, vacuum aspiration, etc.)	1	0	
Manual removal of placenta	1	0	
Special delivery care practices for preventing mother-to-child transmission (PMTCT) of HIV/AIDS	1	0	
Assisted vaginal delivery (apply vacuum or forceps)	1	0	
Resuscitate a newborn with bag and mask (HBB)<br>KMC for low birth weight babies	1	0	
Maternal death or near miss reviews/audits	1	0	
Quality improvement approaches such as standards based management	1	0	
Q114: Have you received additional skilled birth attendant (SBA) training?	1	0	
Q115: In your current position, and as a part of your work for this facility, do you personally provide care for newborns?	1	0	No→Q118
Q116: How many years in total have you provided such services? Service may have been here or in another facility (Observer: enter 00 if less than 1 year of service)	☐☐		
Q117: During the past 3 years have you received any pre- or in-service training on subjects related to newborn care?	1	0	No→Q118
WORKING CONDITIONS IN FACILITY			

Now I would like to ask you some questions about supervision you have personally received. This supervision may have been from a supervisor either in this facility, or from outside the facility. :		
Q118: Do you receive technical support or supervision in your work at this facility? If so, when was the most recent time?		
No, never supervised	0	→Q120
Yes, in the past 3 months	1	
Yes, in the past 4-6 months	2	
Yes, in the past 7-12 months	3	
Yes, more than 12 months ago	4	

Q119: The last time you were personally supervised, did your supervisor do any of the following (read each aloud):	Yes	No
Check your records or reports	1	0
Observe your work	1	0
Give you verbal feedback about how you were doing your job	1	0
Provide any written comment about how you were doing your job	1	0
Provide updates on administrative or technical issues related to your work	1	0
Discuss problems you have encountered	1	0
Participate in quality of care improvement activities	1	0

For question Q120, do not read the answer choices aloud. If you are not sure whether an answer given by health worker matches that listed, probe for more detail. If they give an answer that is not listed, move on to their next answer. use the probe to encourage health worker to give 3 answers. If they cannot give an answer, or give only answers that do not appear in list, circle don't know.

Q120: Among the various things related to your working situation that you would like to see improved, can you tell me the three that you think would most improve your ability to provide good quality of care services? (PROBE: Anything else?)	Code
More support from supervisor	01

More knowledge/ updates / training	02
More supplies/drugs	03
Better quality equipment / supplies	04
Less workload (more staff)	05
Better working hours / flexible times	06
More incentives (salary, promotion, holidays)	07
Increased security	08
Better facility infrastructure	09
More autonomy / independence	10
Emotional support for staff	11
More / better supervision	12
More job aids/guidelines/standards	13
Don't know / None of these	98
End of Section 1	

Section 2: Maternal Health Knowledge Questions

For the following questions, read the question aloud to the health worker. Do not read the answer choices aloud. If you are not sure whether an answer given by health worker matches that listed, probe for more detail. If they give an answer that is not listed, move on to their next answer. use the probe to encourage health worker to give as many answers as they can think of. If they cannot give an answer, or give only answers that do not appear in list, circle don't know.

READ ALOUD: Please answer the following questions on maternal health to the best of your knowledge. Most of the questions I ask you will require multiple responses from you. Assume all needed supplies, medications, and equipment are available. When thinking about your answers, you should include actions or interventions that could be done at your facility and at a referral facility. I will probe sometimes to help you remember some more information. Please provide all responses that come to mind.

Question		Code
Q200: What actions during labor and delivery would you take in an HIV+ woman to prevent/ reduce mother-to-child transmission of the virus?		
	PMTCT counseling	01
(PROBE: Any other actions or interventions?)	Provide ARV prophylaxis to woman in early labor	02
	Wipe nose, mouth, eyes of newborn with gauze, suction only if necessary	03
	No routine episiotomy	04
	Minimize instrument delivery	05
	Hibitane vaginal cleansing	06
	Minimize vaginal exam	07
	Minimize artificial rupture of membranes	08
	Avoid milking cord/ immediate clamp cord	09
	Appropriate use of partograph	10
	Active mgt of 3rd stage labor	11
	Provide ARV prophylaxis to infant	12
	Don't know	98
Q201: What are the key steps for performing active management of the third stage of labor?		

	Administration of a uterotonic immediately/ within 1 minute of delivery	01
(PROBE: if health worker mentions uterotonic, ask when should uterotonic be given?)	Administration of a uterotonic with delivery of anterior shoulder	02
	Administration of a uterotonic after delivery of placenta	03
	Controlled cord traction	04
	Uterine massage	05
	Don't know	98
Q202: What actions are appropriate for a woman who presents with, or develops heavy bleeding postpartum from atonic/uncontracted uterus?		
	Massage the fundus	01
(PROBE: Any other actions or interventions?)	Empty urinary bladder	02
	Give uterotonics IM or IV	03
	Perform bimanual compression of uterus	04
	Perform abdominal compression of aorta	05
	Start IV fluids	06
	Take blood for hb, grouping and x-matching	07
	Insert condom tamponade	08
	Refer to doctor or hospital	09
	Raise foot of bed	10
	Don't know	98
Q203: When should membranes be ruptured artificially by the provider?		
	At start of second stage	01
(PROBE: Any other times)?	Immediately prior to delivery when they are bulging in vagina	02
	Routinely during active phase of labor	03
	As part of augmentation of labor	04
	Upon admission for all women	05
	To check color of fluid/liquor when fetal distress is noted	06
	Not to be ruptured	07
	Don't know	98
Q204: What actions do you believe are most appropriate in managing a woman with severe pre-eclampsia at term?		
	Provide magnesium sulphate	01

(PROBE: Any other actions?)	Provide diazepam	02		
	Provide anti-hypertensives	03		
	Prepare to deliver within 24 hours	04		
	Don't know	98		
Q205: Which antibiotic is given after delivery?				
	Ampicillin	01		
(PROBE: Anything else?)	Gentamicin	02		
	Metronidazole	03		
	Other antibiotic (specify) _________	04		
	Don't know	98		
READ ALOUD: Now I would like to present you with a scenario you might encounter in your practice.<br>A woman is brought to the emergency department of the district hospital by her husband after she complained of a severe headache and blurred vision. She is 20 years old, this is her first pregnancy, and she is 37 weeks gestation. She had 2 ANC visits and no problems. She denies upper abdominal pain or decreased urine output, and fetal movement is normal. Her BP is 160/120. Her examination is normal. She has contractions 2 in 10 minutes, lasting 20 seconds by palpation. Her urine has 3+ protein.				
Q206: Given the information presented above, what is your working diagnosis? (do not read answers aloud)				
	Kidney infection	1		
(CIRCLE ONLY 1 ANSWER)	Severe pre-eclampsia	2		
	Malaria	3		
	Eclampsia	4		
	In labor	5		
	Don't know	98		
For question Q207, read the question aloud to the health worker and then read each procedure aloud.				
Question		Yes	No	DK
Q207: Of the list of procedures I am going to read you, please tell me which procedures are carried out routinely for all patients during labor and delivery at your facility:				
	Artificial rupture of membranes	1	0	8
	Active management of third stage of labor	1	0	8
	Episiotomy	1	0	8
	Perineal shaving	1	0	8
	Maternal blood pressure monitoring	1	0	8

	Administration of prophylactic antibiotics to women in labor	1	0	8
	Enema	1	0	8
	Suctioning nose and mouth of newborn	1	0	8
	Fetal heart rate monitoring	1	0	8
End of Section 2				

Section 3: Newborn Health Knowledge Questions

For the following questions, read the question aloud to the health worker. Do not read the answer choices aloud. If you are not sure whether an answer given by health worker matches that listed, probe for more detail. If they give an answer that is not listed, move on to their next answer. use the probe to encourage health worker to give as many answers as they can think of. If they cannot give an answer, or give only answers that do not appear in list, circle don't know.

READ ALOUD: Please answer the following questions on newborn health to the best of your knowledge. Most of the questions I will be asking you will require multiple responses from you. Assume all needed supplies, medications, and equipment are available. When thinking about your answers, you should include actions or interventions that could be done at your facility and at a referral facility. I will probe sometimes to help you remember some more information. Please provide all responses that come to mind.

Question		Code
Q301: What basic equipment and supplies must be available to ensure the baby receives appropriate immediate care after birth?		
	Dry warm towels or cloths	01
(PROBE: Anything else?)	Sterile blade or scissors	02
	Sterile or disposable cord ties/ clamps	03
	Cap for baby	04
	Source of warmth: heating lamp or incubator	05
	Self-inflating ventilation bag	06
	Newborn face mask size 1	07
	Newborn face mask size 0	08
	Mucus extractor/ simple suction/ bulb syringe	09
	Flat surface	10
	Clock or watch with seconds	11
	Don't know	98

Q302: Please tell me, when a baby is delivered and there is no complication, what care is important to give them immediately after birth and in the first hour?		
	Wipe face after birth of head	01
(PROBE: Anything else?)	Ensure baby was breathing/ crying	02
	Provide thermal protection (skin to skin)	03
	Suction newborn with bulb	04
	Ensure mother initiates breast feeding within 1 hour	05
	Assess/examine newborn within 1 hour	06
	Weigh newborn	07
	Provide eye prophylaxis /antibiotic ointment	08
	Give prelacteal feed/ water	09
	Cut cord with sterile blade/scissors	10
	Apply antiseptic or other material to cord stump	11
	Don't know	98
Q303: Can you please tell me the signs and symptoms of severe infection (sepsis) in a newborn?	Poor/ no breastfeeding	01
(PROBE: Any other signs or symptoms?)	Restlessness/irritability	02
	Breathing difficulties	03
	Hypothermia	04
	Hyperthermia	05
	Breathing rating >60/minute	06
	Convulsions	07
	Pus/ redness around umbilicus	08
	Abscess on any part of body	09
	Skin pustules	10
	Lethargy/ no movement (conscious)	11
	Unconscious	12
	Don't know	98
End of Section 3		
END of Interview		
