# Supplementary material for: Assessment of facility and health worker readiness to provide quality antenatal, intrapartum and postpartum care in rural Southern Nepal
Source: BMC Health Serv Res. 2020 Jan 6;20:16. doi: 10.1186/s12913-019-4871-x (PMC6945781; doi:10.1186/s12913-019-4871-x)
Supplement: Supplementary file 2 — Additional file 2: Labor/delivery room and ANC examination room setting. This additional file includes Table S1. which shows the results on the setting of the labor/delivery room and ANC examination room and basic infrastructure in those rooms. [file 12913_2019_4871_MOESM2_ESM.docx]

**Additional file** **2: Labor/delivery room and ANC examination room setting**

## **Table S1. Setting and infrastructure of the labor/delivery room and ANC room**

|  | **PHCC**  **(N=5)** | **HP**  **(N=16)** | **Private**  **(N=2)** | **District Hospital**  **(N=1)** | **Total**  **(N=24)** |
| --- | --- | --- | --- | --- | --- |
| **Labor and Delivery room** | | | | | |
| **Delivery room setting** |  |  |  |  |  |
| Private room with visual and auditory privacy | 4 | 14 | 2 | 1 | 21 |
| **Condition of the Delivery room** |  |  |  |  |  |
| Clean | 4 | 15 | 2 | 1 | 22 |
| Dirty | 1 | 1 | 0 | 0 | 2 |
| **Toilet for client use, near the delivery room** |  |  |  |  |  |
| Yes, attached to the delivery room | 2 | 2 | 1 | 0 | 5 |
| Yes, but not attached to the delivery room | 3 | 14 | 1 | 1 | 19 |
| Toilet functioning (clean and water available) | 2 | 13 | 2 | 1 | 18 |
| **Other elements to support quality*** |  |  |  |  |  |
| 24hr coverage for deliveries (staff present or on-call, schedule Observed or reported but not seen ) | 5 | 15 | 2 | 1 | 23 |
| Guidelines for normal delivery | 3 | 12 | 0 | 1 | 16 |
| Guidelines for emergency obstetric care | 3 | 12 | 0 | 1 | 16 |
| Blank partographs | 5 | 16 | 2 | 1 | 24 |
| All elements available | 3 | 12 | 0 | 1 | 16 |
| **ANC examination room** |  |  |  |  |  |
| **ANC examination room setting** |  |  |  |  |  |
| Private room with visual and auditory privacy | 4 | 14 | 2 | 1 | 21 |
| Non private room with visual and auditory privacy | 1 | 1 | 0 | 0 | 2 |
| Visual privacy only | 0 | 1 | 0 | 0 | 1 |
| **Condition of the ANC examination room** |  |  |  |  |  |
| Clean | 5 | 13 | 2 | 1 | 21 |
| Dirty | 0 | 3 | 0 | 0 | 3 |
| **Toilet for client use near the ANC service area** | 5 | 16 | 2 | 1 | 24 |
| **Toilet is functioning (clean, water availability/bucket)** | 3 | 13 | 2 | 1 | 19 |
| **Waste receptacle with plastic liner** |  |  |  |  |  |
| No waste bin | 1 | 1 | 0 | 0 | 2 |
| Waste bin with neither lid nor plastic liner | 3 | 8 | 1 | 1 | 13 |
| Waste bin with lid but no plastic liner | 1 | 7 | 0 | 0 | 8 |

**Observed or reported not seen AND functioning*

*†Observed in the ANC, delivery or waiting area of the health facility*
