# Supplementary material for: Assessment of facility and health worker readiness to provide quality antenatal, intrapartum and postpartum care in rural Southern Nepal
Source: BMC Health Serv Res. 2020 Jan 6;20:16. doi: 10.1186/s12913-019-4871-x (PMC6945781; doi:10.1186/s12913-019-4871-x)
Supplement: Supplementary file 3 — Additional file 3: Capacity to perform BEmONC signal functions and immediate newborn care. This additional file includes Table S2., which shows the detail breakdown of the availability of the medicines and supplies to perform each of the seven BEmONC signal functions as well as the availability of medicines/supplies to deal with common or serious complications and immediate newborn care. [file 12913_2019_4871_MOESM3_ESM.docx]

**Additional File 3. Capacity to perform BEmONC signal functions and immediate newborn care**

**Table S2. Availability of the medicines and supplies required to perform the BEmONC signal functions**

| **Medicines and Supplies to perform various BEmONC signal functions*** | **District Hospital**  **(N=1)** | **PHCC**  **(N=5)** | | **HP**  **(N=16)** | **Private**  **(N=2)** | **Total**  **(N=24)** |  |
| --- | --- | --- | --- | --- | --- | --- | --- |
| **Medicine and supplies for common complications** |  |  | |  |  |  |  |
| Syringes | 1 | 5 | | 15 | 2 | 23 |  |
| Needles | 1 | 5 | | 15 | 2 | 23 |  |
| Injectable oxytocin 1IU/mL | 1 | 5 | | 15 | 2 | 23 |  |
| Intravenous solutions | 1 | 5 | | 16 | 2 | 24 |  |
| IV Cannula (1 set) | 1 | 5 | | 15 | 2 | 23 |  |
| Perinea/vaginal/cervical repair set | 1 | 4 | | 11 | 2 | 18 |  |
| *All meds & supplies for common complications* | *1* | *4* | | *10* | *2* | *17* |  |
| **Medicine and supplies for serious complications** |  |  | |  |  |  |  |
| Injectable anticonvulsant (magnesium sulfate or  diazepam) | 1 | 3 | | 8 | 1 | 13 |  |
| Injectable antibiotic (ampicillin or gentamicin) | 1 | 3 | | 9 | 2 | 15 |  |
| *All serious complications supplies present* | 1 | 3 | | 4 | 1 | 9 |  |
| **Assisted Delivery** |  |  | |  |  |  |  |
| Ventouse (Vacuum extractor manual or electrical) | 1 | 0 | | 11 | 1 | 13 |  |
| *Percent Score for assisted delivery* | *100%* | *0%* | | *68.8%* | *50%* | *54.2%* |  |
| **Removal of retained products of conception** |  |  | |  |  |  |  |
| Manual/electric vacuum aspirator or dilation and curettage kit | 1 | 2 | | 2 | 2 | 7 |  |
| Injectable oxytocin | 1 | 5 | | 15 | 2 | 23 |  |
| Syringes and needles | 1 | 5 | | 15 | 2 | 23 |  |
| Ringer's lactate, D5NS or NS infusion | 1 | 5 | | 16 | 2 | 24 |  |
| *Mean percent score for removal of retained products or conception* | *100%* | *85%* | | *75%* | *100%* | *79.2%* |  |
| **Use of parenteral antibiotics for infection** |  |  | |  |  |  |  |
| Injectable ampicillin or gentamicin | 1 | 3 | | 9 | 2 | 15 |  |
| Syringes and needles | 1 | 5 | | 15 | 2 | 23 |  |
| Ringer's lactate, D5NS or NS infusion | 1 | 5 | | 16 | 2 | 24 |  |
| *Mean percent score for parenteral antibiotics use* | *100%* | *86.7%* | | *83.4%* | *100%* | *86.1%* |  |
| **Use of parenteral oxytocic drugs** |  |  | |  |  |  |  |
| Injectable oxytocin | 1 | 5 | | 15 | 2 | 23 |  |
| Syringes and needles | 1 | 5 | | 15 | 2 | 23 |  |
| Ringer's lactate, D5NS or NS infusion | 1 | 5 | | 16 | 2 | 24 |  |
| *Mean percent score for parenteral oxytocin use* | *100%* | *100%* | | *93.8%* | *100%* | *95.8%* |  |
| **Use of parenteral anti-convulsants for PE/E** |  |  | |  |  |  |  |
| Injectable magnesium sulfate | 1 | 3 | | 9 | 1 | 15 |  |
| Syringes and needles | 1 | 5 | | 15 | 2 | 23 |  |
| Ringer's lactate, D5NS or NS infusion | 1 | 5 | | 16 | 2 | 24 |  |
| *Mean percent score for parenteral anti-convulsant*s | *100%* | *86.7%* | | *81.3%* | *83.3%* | *82%* |  |
| **Manual Removal of Placenta** |  |  | |  |  |  |  |
| Injectable ampicillin | 1 | 2 | | 9 | 2 | 13 |  |
| Injectable oxytocin | 1 | 5 | | 15 | 2 | 23 |  |
| Syringes and needles | 1 | 5 | | 15 | 2 | 23 |  |
| Ringer's lactate, D5NS or NS infusion | 1 | 5 | | 16 | 2 | 24 |  |
| *Mean percent score for MRP* | *100%* | *85%* | | *86%* | *100%* | *86.4%* |  |
| **Newborn resuscitation** |  |  | |  |  |  |  |
| Bag/tube and mask (infant size) | 1 | 5 | | 14 | 2 | 22 |  |
| Foot suction or delee suction or electric suction‡ | 1 | 4 | | 12 | 1 | 18 |  |
| Suction apparatus for use with catheter | 0 | 0 | | 2 | 1 | 3 |  |
| Resuscitation table for newborn | 1 | 4 | | 11 | 2 | 18 |  |
| *Mean percent score for newborn resuscitation* | *75%* | *65%* | | *61%* | *75%* | *63.5%* |  |
| **Supplies for immediate newborn care *** |  | |  |  |  |  | |
| Sterile scissors or blade | 1 | | 5 | 16 | 2 | 24 | |
| Sterile cord clamp or tie | 1 | | 5 | 13 | 2 | 21 | |
| Suction machine (Foot or electric or delees suction) | 1 | | 4 | 12 | 1 | 18 | |
| Towel or blanket to wrap baby | 1 | | 4 | 15 | 2 | 22 | |
| Heat source for premature infant | 1 | | 1 | 6 | 1 | 9 | |
| Chlorhexidine (CHX) | 1 | | 5 | 12 | 2 | 20 | |
| Resuscitation table for baby | 1 | | 4 | 11 | 2 | 18 | |
| Bag and mask (infant size) for resuscitation | 1 | | 5 | 14 | 2 | 22 | |
| Oxygen cylinder | 1 | | 2 | 7 | 2 | 12 | |
| Oral thermometer | 1 | | 3 | 9 | 2 | 15 | |

**Observed or reported not seen and functioning*
