# Supplementary material for: Assessment of facility and health worker readiness to provide quality antenatal, intrapartum and postpartum care in rural Southern Nepal
Source: BMC Health Serv Res. 2020 Jan 6;20:16. doi: 10.1186/s12913-019-4871-x (PMC6945781; doi:10.1186/s12913-019-4871-x)
Supplement: Supplementary file 4 — Additional file 4: Health worker training and work experience. This additional file shows the detailed breakdown on the type and years of training the health workers have received along with their number of years of experience working in ANC, delivery care and newborn care. Table S3A. and S3B. displays the results on training and work experience of the health workers by health facility type and SBA training respectively. [file 12913_2019_4871_MOESM4_ESM.docx]

**Additional File 4. Health worker training and work experience**

**Table S3A. Distribution of training and experience working in ANC, delivery care and newborn care by type of health facility**

| Training and Experience | DH (%) | PHCC (%) | HP (%) | Private (%) | Total (%) |
| --- | --- | --- | --- | --- | --- |
| ANC service and training | **N=1** | **N=13** | **N=33** | **N=6** | **N=53** |
| Years of ANC service provided |  |  |  |  |  |
| 1 year and below | 0 | 15.4 | 21.2 | 16.7 | 18.9 |
| 2-5 years | 0 | 15.4 | 36.4 | 0 | 26.4 |
| 6-10 years | 100 | 30.8 | 18.2 | 16.7 | 22.6 |
| 11 and more years | 0 | 38.5 | 24.2 | 66.8 | 32.1 |
| *Median number of years* | *10 years* | *6 years* | *4 years* | *11.5 years* | *6 years* |
| Received pre or in-service ANC training in past 3 years* |  |  |  |  |  |
| No | 100 | 7.7 | 12.1 | 50 | 17 |
| Yes | 0 | 92.3 | 87.9 | 50 | 83 |
| Delivery care service and training | **N=11** | **N=13** | **N=33** | **N=6** | **N=63** |
| Years of Delivery care service provided* |  |  |  |  |  |
| Less than 1 year | 54.5 | 7.7 | 9.1 | 0 | 15.9 |
| 1-2 years | 18.2 | 15.4 | 33.3 | 16.7 | 25.4 |
| 3-5 years | 9.1 | 30.8 | 36.4 | 0 | 27 |
| 6-10 years | 9.1 | 15.4 | 9.1 | 33.3 | 12.7 |
| 11 years and above | 9.1 | 30.8 | 12.1 | 50 | 19.1 |
| *Median number of years* | *0 year* | *5 years* | *3 years* | *10.5 years* | *3 years* |
| Received pre or in-service Delivery care training in past 3 years* |  |  |  |  |  |
| No | 54.5 | 0 | 15.2 | 83.3 | 25.4 |
| Yes | 45.5 | 100 | 84.8 | 16.7 | 74.6 |
| How often partograph is used in normal vaginal delivery |  |  |  |  |  |
| Never | 0 | 0 | 3.0 | 0 | 1.6 |
| Rarely | 0 | 0 | 0 | 0 | 0 |
| Sometimes | 9.1 | 0 | 0 | 0 | 1.6 |
| Most of the time | 9.1 | 0 | 15.2 | 0 | 9.5 |
| Always | 81.8 | 100 | 81.8 | 100 | 87.3 |
| How often Active Management of Third Stage of Labor (AMTSL) used in normal vaginal delivery |  |  |  |  |  |
| Never | 0 | 0 | 0 | 0 | 0 |
| Rarely | 0 | 0 | 3.0 | 0 | 1.6 |
| Sometimes | 0 | 0 | 3.0 | 16.7 | 3.2 |
| Most of the time | 0 | 0 | 6.1 | 33.3 | 6.4 |
| Always | 100 | 100 | 87.9 | 50 | 88.9 |
| Newborn Care service and training | **N=11** | **N=13** | **N=33** | **N=6** | **N=63** |
| Years of Newborn care service provided* |  |  |  |  |  |
| Less than 1 year | 45.4 | 7.7 | 9.1 | 0 | 14.3 |
| 1-2 years | 18.2 | 15.4 | 33.3 | 16.7 | 25.4 |
| 3-5 years | 18.2 | 23.1 | 36.4 | 0 | 27 |
| 6-10 years | 9.1 | 23.1 | 6.1 | 33.3 | 13 |
| 11 years and above | 9.1 | 30.8 | 15.1 | 50 | 20.6 |
| *Median number of years* | *1 year* | *6 years* | *3 years* | *10.5 years* | *4 years* |
| Received pre or in-service Newborn care training in past 3 years |  |  |  |  |  |
| No | 54.5 | 23.1 | 27.3 | 66.7 | 34.9 |
| Yes | 45.5 | 76.9 | 72.7 | 33.3 | 65.1 |

**Fishers exact test p-value < 0.05*

**Table S3B. Distribution of training and experience working in ANC, delivery care and newborn care by health worker who received and did not receive additional SBA training**

| Training and Experience | Non-SBA trained (%) | SBA trained (%) | Total (%) |
| --- | --- | --- | --- |
| ANC service and training | N=24 | N=29 | N=53 |
| Years of ANC service provided |  |  |  |
| 1 year and below | 29.2 | 10.3 | 18.9 |
| 2-5 years | 29.2 | 24.1 | 26.4 |
| 6-10 years | 16.7 | 27.6 | 22.6 |
| 11 and more years | 25 | 37.9 | 32.1 |
| *Median number of years* | *3.5 years* | *7 years* | *6 years* |
| Received pre or in-service ANC training in past 3 years |  |  |  |
| No | 20.8 | 13.8 | 17 |
| Yes | 79.2 | 86.2 | 83 |
| Delivery Care Service and Training | N=31 | N=32 | N=63 |
| Years of Delivery care service provided |  |  |  |
| Less than 1 year | 25.8 | 6.3 | 10 (15.9%) |
| 1-2 years | 32.3 | 18.8 | 16 (25.4%) |
| 3-5 years | 22.6 | 31.3 | 17 (27%) |
| 6-10 years | 6.5 | 18.8 | 8 (12.7%) |
| 11 years and above | 12.9 | 25 | 12 (19%) |
| *Median number of years* | *2 years* | *5 years* | *3 years* |
| Received pre or in-service Delivery care training in past 3 years |  |  |  |
| No | 41.9 | 9.4 | 25.4) |
| Yes | 58.1 | 90.6 | 74.6 |

| **How often partograph is used in normal vaginal delivery** | N=31 | N=32 | N=63 |
| --- | --- | --- | --- |
| Never | 3.2 | 0 | 1.6 |
| Rarely | 0 | 0 | 0 |
| Sometimes | 0 | 3.1 | 1.6 |
| Most of the time | 6.5 | 12.5 | 9.5 |
| Always | 90.3 | 84.4 | 87.3 |
| **How often Active Management of Third Stage of Labor (AMTSL) used in normal vaginal delivery** |  |  |  |
| Never | 0 | 0 | 0 |
| Rarely | 3.2 | 0 | 1.6 |
| Sometimes | 6.5 | 0 | 3.2 |
| Most of the time | 6.5 | 6.3 | 6.3 |
| Always | 83.9 | 93.8 | 88.9 |
| **Newborn care service and training** | N=31 | N=32 | N=63 |
| **Years of Newborn care service provided** |  |  |  |
| Less than 1 year | 19.4 | 9.4 | 14.3 |
| 1-2 years | 35.5 | 15.6 | 25.4 |
| 3-5 years | 22.6 | 31.3 | 27 |
| 6-10 years | 9.7 | 15.6 | 12.7 |
| 11 years and above | 12.9 | 28.1 | 20.6 |
| ***Median number of years*** | *2 years* | *5 years* | *4 years* |
| **Received pre or in-service Newborn care training in past 3 years*** | N=31 | N=32 | N=63 |
| No | 51.6 | 18.8 | 34.9 |
| Yes | 48.4 | 81.3 | 65.1 |

**Significant difference at Chi square test p-value=0.006*
