# Supplementary material for: Assessment of facility and health worker readiness to provide quality antenatal, intrapartum and postpartum care in rural Southern Nepal
Source: BMC Health Serv Res. 2020 Jan 6;20:16. doi: 10.1186/s12913-019-4871-x (PMC6945781; doi:10.1186/s12913-019-4871-x)
Supplement: Supplementary file 5 — Additional file 5: Health worker knowledge on maternal health. This additional file shows the detailed breakdown on health worker knowledge on various topics of maternal health by type of health facility (Table S4A.) and by SBA training (Table S4B). [file 12913_2019_4871_MOESM5_ESM.docx]

**Additional File 5: Health worker knowledge on maternal health**

**Table S4A. Distribution of knowledge of key topics of maternal health among health workers by type of health facility**

| Maternal Health Knowledge Topics | DH (%)  N=11 | PHCC (%)  N=13 | HP (%)  N=33 | Private (%)  N=6 | Total (%)  N=63 |
| --- | --- | --- | --- | --- | --- |
| Actions to be taken to reduce PMTCT during labor & delivery |  |  |  |  |  |
| PMTCT counseling | 18.2 | 0 | 6.1 | 0 | 6.3 |
| Provide ARV prophylaxis to woman in early labor* | 36.4 | 0 | 9.1 | 16.7 | 12.7 |
| Wipe nose, mouth, eyes of newborn with gauze, suction only if necessary | 0 | 7.7 | 3 | 0 | 3.2 |
| No routine episiotomy* | 27.3 | 0 | 3 | 16.7 | 7.9 |
| Minimize instrument delivery | 18.2 | 15.4 | 3 | 0 | 7.9 |
| Hibitane vaginal cleansing | 0 | 0 | 0 | 0 | 0 |
| Minimize vaginal exam | 0 | 15.4 | 0 | 0 | 3.2 |
| Minimize artificial rupture of membranes | 0 | 15.4 | 6.1 | 0 | 6.3 |
| Avoid milking cord/ immediate clamp cord | 9.1 | 30.8 | 24.2 | 0 | 20.6 |
| Appropriate use of partograph | 0 | 0 | 0 | 0 | 0 |
| Active mgt of 3rd stage labor | 0 | 7.7 | 0 | 0 | 1.6 |
| Provide ARV prophylaxis to infant | 27.3 | 0 | 15.2 | 16.7 | 14.3 |
| Don’t know | 27.3 | 53.8 | 57.6 | 83.3 | 54 |
| *Mean Percent Score* | 11.4 | 7.7 | 5.8 | 4.2 | 7 |
| Key steps for performing AMTSL |  |  |  |  |  |
| Administration of a uterotonic immediately/ within 1 minute of delivery | 100 | 100 | 97 | 66.7 | 95.2 |
| Controlled cord traction | 90.9 | 92.3 | 93.9 | 66.7 | 90.5 |
| Uterine massage* | 100 | 100 | 93.9 | 50 | 92.1 |
| All three components of AMTSL* | 90.9 | 92.3 | 90.9 | 33.3 | 85.7 |
| When should artificial rupture of membranes be done by the provider |  |  |  |  |  |
| *To check color of fluid/liquor when fetal distress is noted** | 36.4 | 23.1 | 6.1 | 0 | 14.3 |
| As part of augmentation of labor | 36.4 | 15.4 | 36.4 | 0 | 28.6 |
| *Mean percent score* | 36.4 | 19.25 | 21.25 | 0 | 21.4 |
| *WRONG: At start of second stage** | 63.6 | 23.1 | 69.7 | 2 | 55.6 |
| WRONG: Immediately prior to delivery when they are bulging in vagina | 54.5 | 84.6 | 48.5 | 4 | 58.7 |
| WRONG: Routinely during active phase of labor | 0 | 0 | 0 | 1 | 1.6 |
| WRONG: Upon admission for all women | 9.1 | 0 | 0 | 0 | 1.6 |
| WRONG: Not to be ruptured | 0 | 7.7 | 3 | 0 | 3.2 |
| Don't know | 9.1 | 0 | 0 | 0 | 1.6 |
| Actions appropriate for heavy bleeding postpartum from atonic / uncontracted uterus |  |  |  |  |  |
| Massage the fundus | 90.9 | 92.3 | 84.8 | 50 | 84.1 |
| Empty urinary bladder | 45.5 | 7.7 | 39.4 | 50 | 34.9 |
| Give uterotonics IM or IV | 100 | 92.3 | 93.9 | 100 | 95.2 |
| *Perform bimanual compression of uterus* | 27.3 | 38.5 | 9.1 | 33.3 | 20.6 |
| Perform abdominal compression of aorta | 18.2 | 38.5 | 21.2 | 0 | 22.2 |
| Start IV fluids | 100 | 84.6 | 84.8 | 83.3 | 87.3 |
| Take blood for hb, grouping and x-matching | 9.1 | 7.7 | 6.1 | 0 | 6.3 |
| Insert condom tamponade | 9.1 | 0 | 0 | 0 | 1.6 |
| Refer to doctor or hospital | 18.2 | 38.5 | 30.3 | 33.3 | 30.2 |
| Raise foot of bed | 0 | 0 | 9.1 | 0 | 4.8 |
| *Mean Score Percent* | *41.83* | *40.01* | *37.87* | *35* | *38.7* |
| Actions most appropriate in managing a woman with severe pre-eclampsia at term |  |  |  |  |  |
| Provide magnesium sulphate | 90.9 | 100 | 75.8 | 66.7 | 82.5 |
| Provide diazepam | 9.1 | 7.7 | 15.2 | 33.3 | 14.3 |
| Provide anti-hypertensives | 54.5 | 38.5 | 42.4 | 50 | 44.4 |
| Prepare to deliver within 24 hours | 63.6 | 38.5 | 27.3 | 0 | 33.3 |
| Don't know | 9.1 | 0 | 9.1 | 0 | 6.4 |
| *Mean Score Percent (excluding diazepam and DK)* | *69.7* | *59.0* | *48.5* | *30* | *36.2* |
| Antibiotics used after delivery |  |  |  |  |  |
| Ampicillin | 63.6 | 30.8 | 12.1 | 0 | 23.8 |
| Metronidazole | 9.1 | 30.8 | 27.3 | 0 | 22.2 |
| Amoxicilin | 63.6 | 76.9 | 69.7 | 66.7 | 69.8 |
| Other antibiotic | 54.5 | 46.2 | 66.7 | 33.3 | 57.1 |
| Don't know | 0 | 0 | 3 | 0 | 1.6 |
| Diagnosis to the case of pre-eclampsia * |  |  |  |  |  |
| Severe pre-eclampsia | 90.9 | 100 | 63.6 | 16.7 | 71.4 |
| Eclampsia | 9.1 | 0 | 33.3 | 66.7 | 25.4 |
| Don’t Know | 0 | 0 | 3.0 | 16.6 | 3.2 |

**Fishers exact test p-value < 0.05*

**Table S4B. Distribution of knowledge of maternal health among health workers by health worker who received and did not receive additional SBA training**

| Maternal Health Knowledge Topics | Non-SBA trained (%)  N=31 | SBA trained (%)  N=32 | Total (%)  N=63 |
| --- | --- | --- | --- |
| Actions to be taken to reduce PMTCT during labor & delivery |  |  |  |
| PMTCT counseling | 2 (6.5%) | 2 (6.3%) | 4 (6.3%) |
| Provide ARV prophylaxis to woman in early labor* | 1 (3.2%) | 7 (21.9%) | 8 (12.7%) |
| Wipe nose, mouth, eyes of newborn with gauze, suction only if necessary | 1 (3.2%) | 1 (3.1%) | 2 (3.2%) |
| No routine episiotomy | 2 (6.5%) | 3 (9.4%) | 5 (7.9%) |
| Minimize instrument delivery | 3 (9.7%) | 2 (6.3%) | 5 (7.9%) |
| Hibitane vaginal cleansing | 0 (0%) | 0 (0%) | 0 (0%) |
| Minimize vaginal exam | 0 (0%) | 2 (6.3%) | 2 (3.2%) |
| Minimize artificial rupture of membranes | 1 (3.2%) | 3 (9.4%) | 4 (6.3%) |
| Avoid milking cord/ immediate clamp cord | 9 (29%) | 4 (12.5%) | 13 (20.6%) |
| Appropriate use of partograph | 0 (0%) | 0 (0%) | 0 (0%) |
| Active mgt of 3rd stage labor | 1 (3.2%) | 0 (0%) | 1 (1.6%) |
| Provide ARV prophylaxis to infant | 2 (6.5%) | 7 (21.9%) | 9 (14.3%) |
| Don’t know | 16 (51.6%) | 18 (56.3%) | 34 (54%) |
| *Mean Percent Score* | *5.9%* | *8.1 %* | *7%* |
| Key steps for performing AMTSL |  |  |  |
| Administration of a uterotonic immediately/ within 1 minute of delivery | 28 (90.3%) | 32 (100%) | 60 (95.2%) |
| Controlled cord traction | 28 (90.3%) | 29 (90.6%) | 57 (90.5%) |
| *Uterine massage†* | *26 (83.9%)* | *32 (100%)* | *58 (92.1%)* |
| Don't know | 1 (3.2%) | 0 (0%) | 1 (1.6%) |
| All three components of AMTSL | 25 (80.6%) | 29 (90.6%) | 54 (85.7%) |
| When should artificial rupture of membranes be done by the provider |  |  |  |
| To check color of fluid/liquor when fetal distress is noted | 4 (12.9%) | 5 (15.6%) | 9 (14.3%) |
| As part of augmentation of labor | 6 (19.4%) | 12 (37.5%) | 18 (28.6%) |
| *Mean percent score* | 16.15% | 26.55% | 21.45% |
| WRONG: At start of second stage | 14 (452%) | 21 (65.6%) | 35 (55.6%) |
| *WRONG: Immediately prior to delivery when they are bulging in vagina‡* | *23 (74.2%)* | *14 (43.8%)* | *37 (58.7%)* |
| WRONG: Routinely during active phase of labor | 0 (0%) | 1 (3.2%) | 1 (1.6%) |
| WRONG: Upon admission for all women | 1 (3.2%) | 0 (0%) | 1 (1.6%) |
| WRONG: Not to be ruptured | 1 (3.2%) | 1 (3.2%) | 2 (3.2%) |
| Don't know | 1 (3.2%) | 0 (0%) | 1 (1.6%) |
| Actions appropriate for heavy bleeding postpartum from atonic / uncontracted uterus |  |  |  |
| Massage the fundus ^††^ | 23 (74.2%) | 30 (93.8%) | 53 (84.1%) |
| Empty urinary bladder | 8 (25.8%) | 14 (43.8%) | 22 (34.9%) |
| Give uterotonics IM or IV | 30 (96.8%) | 30 (93.8%) | 60 (95.2%) |
| Perform bimanual compression of uterus | 4 (12.9%) | 9 (28.1%) | 13 (20.6%) |
| *Perform abdominal compression of aorta^††^* | *3 (9.7%)* | *11 (34.4%)* | *14 (22.2%)* |
| Start IV fluids | 27 (87.1%) | 28 (87.5%) | 55 (87.3%) |
| Take blood for hb, grouping and x-matching | 2 (6.5%) | 2 (6.3%) | 4 (6.3%) |
| Insert condom tamponade | 0 (0%) | 1 (3.1%) | 1 (1.6%) |
| Refer to doctor or hospital | 9 (29%) | 10 (31.3%) | 19 (30.2%) |
| Raise foot of bed | 0 (0%) | 3 (9.4%) | 3 (4.8%) |
| *Mean Score Percent* | *34.20%* | *43.15%* | *38.72%* |
| Actions most appropriate in managing a woman with severe pre-eclampsia at term |  |  |  |
| *Provide magnesium sulphate†* | *22 (71%)* | *30 (93.8%)* | *52 (82.5%)* |
| Provide diazepam | 5 (16.1%) | 4 (12.5%) | 9 (14.3%) |
| Provide anti-hypertensives | 13 (41.9%) | 15 (46.9%) | 28 (44.4%) |
| Prepare to deliver within 24 hours | 7 (22.6%) | 14 (43.8%) | 21 (33.3%) |
| Don't know | 4 (12.9%) | 0 (0%) | 4 (6.3%) |
| *Mean Score Percent (excluding diazepam)* | *45.20%* | *61.50%* | *53.40%* |
| Antibiotics used after delivery |  |  |  |
| Ampicillin | 6 (19.4%) | 9 (28.1%) | 15 (23.8%) |
| Metronidazole | 6 (19.4%) | 8 (25%) | 14 (22.2%) |
| Amoxicilin | 21 (67.7%) | 23 (71.9%) | 44 (69.8%) |
| Other antibiotic | 17 (54.8%) | 19 (59.4%) | 36 (57.1%) |
| Don't know | 1 (3.2%) | 0 (0%) | 1 (1.6%) |
| Diagnosis to the case of pre-eclampsia |  |  |  |
| Severe pre-eclampsia | 19 (61.3%) | 26 (81.3%) | 41 (71.4%) |
| Eclampsia | 10 (32.3%) | 6 (18.7%) | 16 (21.4%) |
| Don’t Know | 2 (6.5%) | 0 (0%) | 2 (3.2%) |
|  |  |  |  |

**Fishers exact test p-value is 0.053; † Fishers exact test p-value is 0.02;* ^††^ Fishers exact test p-value <0.05

*‡ Chi square test p-value = 0.014*
