# Supplementary material for: Assessment of facility and health worker readiness to provide quality antenatal, intrapartum and postpartum care in rural Southern Nepal
Source: BMC Health Serv Res. 2020 Jan 6;20:16. doi: 10.1186/s12913-019-4871-x (PMC6945781; doi:10.1186/s12913-019-4871-x)
Supplement: Supplementary file 6 — Additional file 6: Health worker knowledge on newborn health. This additional file shows the detailed breakdown on health worker knowledge on various topics of newborn care by type of health facility (Table S5A.) and by SBA training (Table S5B). [file 12913_2019_4871_MOESM6_ESM.docx]

**Additional File 6: Health worker knowledge on newborn health**

**Table S5A. Newborn Health Knowledge by type of health facility**

| Newborn Health Knowledge Topics | DH (%)  N=11 | PHCC (%)  N=13 | HP (%)  N=33 | Private (%)  N=6 | Total (%)  N=63 |
| --- | --- | --- | --- | --- | --- |
| Equipment and Supplies considered necessary to ensure appropriate immediate newborn care |  |  |  |  |  |
| Dry warm towels or cloths | 72.7 | 100 | 93.9 | 83.3 | 90.5 |
| Sterile blade or scissors | 36.4 | 23.1 | 33.3 | 16.7 | 30.2 |
| Sterile or disposable cord ties/ clamps | 72.7 | 53.8 | 66.7 | 50 | 63.5 |
| Cap for baby | 0 | 7.7 | 0 | 0 | 1.6 |
| Source of warmth: heating lamp or incubator | 54.5 | 76.9 | 45.5 | 66.7 | 55.6 |
| Self-inflating ventilation bag | 100 | 76.9 | 66.7 | 66.7 | 74.6 |
| Newborn face mask size 1 | 54.5 | 53.8 | 54.5 | 16.7 | 50.8 |
| Newborn face mask size 0 | 45.5 | 38.5 | 54.5 | 0 | 44.4 |
| Mucus extractor/ simple suction/ bulb syringe | 100 | 100 | 81.8 | 83.3 | 88.9 |
| Flat surface | 0 | 7.7 | 24.2 | 0 | 14.3 |
| Clock or watch | 0 | 7.7 | 12.1 | 0 | 7.9 |
| Don't know | 0 | 0 | 3 | 0 | 1.6 |
| *Mean Percent Score* | *48.8* | *49.6* | *48.5* | *31.9* | *43.6* |
| Immediate newborn care after birth and within the first hour for baby delivered with no complications |  |  |  |  |  |
| Wipe face after birth of head | 18.2 | 18.2 | 39.4 | 16.7 | 36.5 |
| Ensure baby was breathing/ crying | 72.7 | 72.7 | 42.4 | 33.3 | 46.0 |
| Provide thermal protection (skin to skin) | 63.6 | 63.6 | 75.8 | 83.3 | 79.4 |
| Ensure mother initiates breast feeding within 1 hour | 100 | 100 | 93.9 | 100 | 95.2 |
| Assess/examine newborn within 1 hour | 54.5 | 54.5 | 60.6 | 66.7 | 61.9 |
| Provide eye prophylaxis /antibiotic ointment | 0 | 0 | 6.1 | 50 | 9.5 |
| Cut cord with sterile blade/scissors | 36.4 | 36.4 | 36.4 | 33.3 | 38.1 |
| Apply antiseptic or other material to cord stump | 81.8 | 81.8 | 81.8 | 50 | 76.2 |
| WRONG: Suction newborn with bulb | 9.1 | 9.1 | 18.2 | 66.7 | 25.4 |
| WRONG: Weigh newborn | 27.3 | 27.3 | 54.5 | 33.3 | 47.6 |
| WRONG: Give prelacteal feed/ water | 9.1 | 9.1 | 0 | 0 | 1.6 |
| Mean Percent Score | *53.4* | *53.4* | *54.55* | *54.2* | *55.3* |
| Signs and symptoms of severe newborn infection (sepsis) |  |  |  |  |  |
| Poor/ no breastfeeding | 90.9 | 76.9 | 72.7 | 66.7 | 76.2 |
| *Restlessness/irritability†* | 54.5 | 7.7 | 27.3 | 83.3 | 33.3 |
| *Breathing difficulty* | 54.5 | 69.2 | 39.4 | 50 | 49.2 |
| *Hypothermia†* | 18.2 | 76.9 | 48.5 | 0 | 44.4 |
| Hyperthermia | 90.9 | 92.3 | 78.8 | 66.7 | 82.5 |
| Breathing rating >60/minute | 18.2 | 15.4 | 21.2 | 16.7 | 19.1 |
| Convulsions | 9.1 | 7.7 | 12.1 | 0 | 9.5 |
| Pus/ redness around umbilicus | 63.6 | 61.5 | 69.7 | 16.7 | 61.9 |
| Abscess on any part of body | 0 | 15.4 | 9.1 | 0 | 7.9 |
| *Skin pustules†* | 9.1 | 53.8 | 69.7 | 16.7 | 50.8 |
| Lethargy/ no movement (conscious) | 36.4 | 61.5 | 39.4 | 33.3 | 42.9 |
| Unconscious | 0 | 7.7 | 15.2 | 0 | 9.5 |
| *Mean Percent Score (all)* | *37.1* | *45.5* | *41.9* | *29.2* | *40.6* |
| *Mean percent score (first five symptoms)* | *61.8* | *64.6* | *53.34* | *53.34* | *57.12* |

*‡ Fishers exact test p-value <0.05*

*†* *Fishers exact test p-value < 0.01*

**Table S5B. Newborn health knowledge by health worker who received and did not receive additional SBA training**

| Newborn Health Knowledge Topics | Non-SBA trained (%)  N=31 | SBA trained (%)  N=32 | Total (%)  N=63 |
| --- | --- | --- | --- |
| Equipment and Supplies considered necessary to ensure appropriate immediate newborn care |  |  |  |
| Dry warm towels or cloths | 27 (87.1%) | 30 (93.8%) | 57 (90.5%) |
| Sterile blade or scissors | 10 (32.3%) | 9 (28.1%) | 19 (30.2%) |
| Sterile or disposable cord ties/ clamps | 18 (58.1%) | 22 (68.8%) | 40 (63.5%) |
| Cap for baby | 0 (0%) | 1 (3.1%) | 1 (1.6%) |
| Source of warmth: heating lamp or incubator | 15 (48.4%) | 20 (62.5%) | 35 (55.6%) |
| Self-inflating ventilation bag | 20 (64.5%) | 27 (84.4%) | 47 (74.6%) |
| Newborn face mask size 1 | 12 (38.7%) | 20 (62.5%) | 32 (50.8%) |
| Newborn face mask size 0 | 10 (32.3%) | 18 (56.3%) | 28 (44.4%) |
| Mucus extractor/ simple suction/ bulb syringe | 26 (83.9%) | 30 (93.8%) | 56 (88.9%) |
| Flat surface | 4 (12.9%) | 5 (15.6%) | 9 (14.3%) |
| Clock or watch | 2 (6.5%) | 3 (9.4%) | 5 (7.9%) |
| Don't know | 1 (3.2%) | 0 (0%) | 1 (1.6%) |
| *Mean Percent Score* | *39%* | *48.20%* | *43.70%* |
| Immediate newborn care after birth and within the first hour for baby delivered with no complications |  |  |  |
| Wipe face after birth of head | 13 (41.9%) | 10 (31.3%) | 23 (36.5%) |
| Ensure baby was breathing/ crying | 12 (38.7%) | 17 (53.1%) | 29 (46%) |
| *Provide thermal protection (skin to skin)‡* | *21 (67.7%)* | *29 (90.6%)* | *50 (79.4%)* |
| Ensure mother initiates breast feeding within 1 hour | 29 (93.5%) | 31 (96.8%) | 60 (95.2%) |
| Assess/examine newborn within 1 hour | 16 (51.6%) | 23 (71.9%) | 39 (61.9%) |
| Provide eye prophylaxis /antibiotic ointment | 4 (12.9%) | 2 (6.3%) | 6 (9.5%) |
| Cut cord with sterile blade/scissors | 11 (35.5%) | 13 (40.6%) | 24 (38.1%) |
| Apply antiseptic or other material to cord stump | 22 (71%) | 26 (81.3%) | 48 (76.2%) |
| WRONG: Suction newborn with bulb | 7 (22.6%) | 9 (28.1%) | 16 (25.4%) |
| WRONG: Weigh newborn | 15 (48.4%) | 15 (46.9%) | 30 (47.6%) |
| WRONG: Give prelacteal feed/ water | 1 (3.2%) | 0 (0%) | 1 (1.6%) |
| Mean Percent Score | 51.60% | 59% | 55.40% |
| Signs and symptoms of severe newborn infection (sepsis) |  |  |  |
| Poor/ no breastfeeding | 22 (71%) | 26 (81.3%) | 48 (76.2%) |
| Restlessness/irritability | 14 (45.2%) | 7 (21.9%) | 21 (33.3%) |
| Breathing difficulty | 58.1 | 40.6 | 49.2 |
| *Hypothermia†* | *9 (29%)* | *19 (59.4%)* | *28 (44.4%)* |
| Hyperthermia | 23 (74.2%) | 29 (90.6%) | 52 (82.5%) |
| Breathing rating >60/minute | 5 (16.1%) | 7 (21.9%) | 12 (19%) |
| Convulsions | 2 (6.5%) | 4 (12.5%) | 6 (9.5%) |
| Pus/ redness around umbilicus | 17 (54.8%) | 22 (68.8%) | 39 (61.9%) |
| Abscess on any part of body | 3 (9.7%) | 2 (6.3%) | 5 (7.9%) |
| Skin pustules | 12 (38.7%) | 20 (62.5%) | 32 (50.8%) |
| *Lethargy/ no movement (conscious)††* | *9 (29%)* | *18 (56.3%)* | *27 (42.9%)* |
| Unconscious | 1 (3.2%) | 5 (15.6%) | 6 (9.5%) |
| *Mean Percent Score (all)* | *36.3%* | *44.8%* | *40.6%* |
| *Mean Percent score (first five only)* | *55.5%* | *58.8%* | *57.1%* |

*‡ Fishers exact test p-value =0.03*

*† Chi-square test p-value=0.015*

*†† Chi-square test p-value=0.03*
